# Macroautophagy in lymphatic endothelial cells inhibits T cell–mediated autoimmunity

**DOI:** 10.1084/jem.20201776

**Published:** 2021-04-16

**Authors:** Guillaume Harlé, Camille Kowalski, Juan Dubrot, Dale Brighouse, Gaëlle Clavel, Robert Pick, Natacha Bessis, Jennifer Niven, Christoph Scheiermann, Monique Gannagé, Stéphanie Hugues

**Affiliations:** 1 Department of Pathology and Immunology, School of Medicine, University of Geneva, Geneva, Switzerland; 2 Institut National de la Santé et de la Recherche Médicale, UMR 1125, Université Sorbonne Paris Cité, Université Paris, Paris, France; 3 Service of Immunology and Allergy, Lausanne University Hospital, University of Lausanne, Lausanne, Switzerland

## Abstract

Lymphatic endothelial cells (LECs) present peripheral tissue antigens to induce T cell tolerance. In addition, LECs are the main source of sphingosine-1-phosphate (S1P), promoting naive T cell survival and effector T cell exit from lymph nodes (LNs). Autophagy is a physiological process essential for cellular homeostasis. We investigated whether autophagy in LECs modulates T cell activation in experimental arthritis. Whereas genetic abrogation of autophagy in LECs does not alter immune homeostasis, it induces alterations of the regulatory T cell (T reg cell) population in LNs from arthritic mice, which might be linked to MHCII-mediated antigen presentation by LECs. Furthermore, inflammation-induced autophagy in LECs promotes the degradation of Sphingosine kinase 1 (SphK1), resulting in decreased S1P production. Consequently, in arthritic mice lacking autophagy in LECs, pathogenic Th17 cell migration toward LEC-derived S1P gradients and egress from LNs are enhanced, as well as infiltration of inflamed joints, resulting in exacerbated arthritis. Our results highlight the autophagy pathway as an important regulator of LEC immunomodulatory functions in inflammatory conditions.

## Teaser

The authors show that autophagy in lymphatic endothelial cells (LECs) dampens collagen-induced arthritis by regulating two distinct pathways: the promotion of T reg cells in LNs and the inhibition of S1P-dependent migration of pathogenic Th17 cells in inflamed organs.

## Introduction

LN stromal cells (LNSCs) are CD45^neg^ nonhematopoietic cells that play an important role in LN, blood, and lymphatic vessel architecture. They include three main subsets, the blood endothelial cells (BECs; gp38^−^CD31^+^), the fibroblastic reticular cells (FRCs; gp38^+^CD31^−^), and the lymphatic endothelial cells (LECs; gp38^+^CD31^+^; Rodda et al., 2018). However, LNSC functions are not restricted to their architectural role. In particular, they regulate T cell immunity, notably by altering dendritic cell (DC) maturation and migration (Buettner and Bode, 2011; Podgrabinska et al., 2009). Under inflammatory conditions, DC–LEC interactions lead to a down-regulation of costimulatory molecules by DCs, resulting in their decreased abilities to activate naive T cells (Podgrabinska et al., 2009; Rouhani et al., 2015). In addition, LNSCs ectopically express self-antigens (Ags) restricted to peripheral tissues (also known as peripheral tissue Ags [PTAs]). The direct presentation of PTAs by LNSCs via MHC class I (MHCI) to autoreactive CD8^+^ T cells induces their abortive proliferation and deletion, indicating an important contribution of LNSCs to the maintenance of peripheral CD8^+^ T cell tolerance (Cohen et al., 2010; Fletcher et al., 2010; Gardner et al., 2008; Lee et al., 2007; Magnusson et al., 2008; Nichols et al., 2007; Yip et al., 2009). In addition, the cross-presentation of exogenous Ags by LECs primes a small fraction of naive CD8^+^ T cells into long-lived memory cells capable of quickly acquiring effector functions upon Ag rechallenge (Vokali et al., 2020). At steady state, LNSCs express low basal levels of MHC class II (MHCII) molecules, which are up-regulated upon inflammatory conditions in the presence of IFN-γ (Dubrot et al., 2014). We have shown that LNSCs contribute to CD4^+^ T cell tolerance by presenting peptide–MHCII complexes acquired from DCs to induce CD4^+^ T cell dysfunction (Dubrot et al., 2014). Other studies have described that MHCII expression by LNSCs, especially LECs and FRCs, plays important roles in the homeostasis of regulatory T cells (T reg cells; Nadafi et al., 2020; Baptista et al., 2014), which are crucial for the regulation of (auto)immune cell activation. We recently demonstrated that the abolition of endogenous MHCII expression in LNSCs induces an alteration of T reg cells, leading to the development of spontaneous signs of autoimmunity in elderly mice (Dubrot et al., 2018).

Additional ways for LNSCs to regulate the immune system include the release of soluble factors such as indoleamine 2,3-dioxygenase, nitrite oxide, or CCL21 (Lukacs-Kornek et al., 2011; Malhotra et al., 2012; Nörder et al., 2012; Podgrabinska et al., 2002). Furthermore, LECs are the main source of the messenger sphingolipid sphingosine-1-phosphate (S1P; Pham et al., 2010). S1P is produced after phosphorylation of sphingosine by two isoforms of Sphingosine kinase (SphK), SphK1 and SphK2, localized in the cytosol and in organelles such as the mitochondria and nucleus, respectively (Wattenberg, 2010; Siow et al., 2010). S1P acts through five G protein–coupled receptors (S1P1–5; Pham et al., 2008) and plays an important role in naive T cell survival (Mendoza et al., 2017) and effector T cell trafficking via S1P1 (Garris et al., 2014). Blockade of the S1P signaling pathway using FTY-720 (fingolimod), an S1P receptor antagonist, inhibits T cell egress from LNs, resulting in decreased development of autoimmune diseases such as multiple sclerosis (Seki et al., 2010; Kappos et al., 2006) and rheumatoid arthritis (Tsunemi et al., 2010; Yoshida et al., 2013).

Autophagy is a physiological cellular process playing a major role in the maintenance of cell homeostasis through the degradation of protein aggregates and damaged organelles (Feng et al., 2014). Three main pathways were described, macroautophagy, microautophagy, and chaperone-mediated autophagy, among which macroautophagy (herein referred as “autophagy”) has been the most widely studied. Autophagy is highly regulated by several genes called autophagy-related genes (Atgs) encoding proteins implicated in the formation of autophagosomes. Among these proteins, the complex composed by the proteins Atg5, Atg12, and Atg16L is crucial for the initiation of autophagosome membrane elongation (Feng et al., 2014). The protein light chain 3 (LC3) is a mammalian homologue of Atg8, which is essential for the pathway. Throughout autophagosome generation, the cytosolic microtubule-associated LC3-I is conjugated to phosphatidylethanolamine (PE) to form the LC3-PE (or LC3-II) group, which is recruited to the autophagosome membrane. LC3-interacting regions (LIRs) further facilitate proteins targeting to autophagosomes. Afterward, autophagosomes merge with lysosomes to deliver components for enzymatic degradation (Feng et al., 2014).

Autophagy is implicated in multiple cellular functions, such as cell cycle, lipid metabolism, and Ag presentation (Gannagé and Münz, 2009; Mathiassen et al., 2017; Singh et al., 2009). Several studies revealed an implication of autophagy in the cross-presentation of exogenous Ag through MHCI by DCs in inflammatory contexts such as tumors or viral infection (Li et al., 2009; Loi et al., 2016; Uhl et al., 2009). Moreover, autophagy is implicated in MHCII-restricted Ag presentation. In medullary thymic epithelial cells (mTECs), autophagy contributes to self-Ag loading onto MHCII, therefore participating in the negative selection of auto-reactive CD4^+^ T cells (Aichinger et al., 2013). In DCs, autophagy allows the loading of intracellular viral Ags onto MHCII, thus enhancing viral CD4^+^ T cell responses (Münz, 2009). In experimental autoimmune encephalomyelitis, autophagy deletion in DCs inhibits encephalitogenic CD4^+^ T cell priming and therefore disease development (Bhattacharya et al., 2014; Alissafi et al., 2017). In contrast, autophagy deletion in DCs in arthritic mice impairs their ability to maintain T reg cell functions and stability, leading to their conversion toward T helper 17 (Th17) cells (Niven et al., 2019). Thus, autophagy plays crucial immunomodulatory roles in mTECs and DCs. However, the role of autophagy in LNSCs, which are important actors of peripheral T cell responses, has not been investigated yet.

Here, we investigated whether autophagy in LNSCs affects the development of T cell–mediated autoimmune disease. We demonstrated that in collagen-induced arthritis (CIA), a Th1/Th17-mediated experimental arthritis mouse model, autophagy abrogation in LECs results in decreased T reg cell proliferation and expression of CD103 in inflamed LNs. Together with other studies showing a role for MHCII expression by LECs in promoting T reg cell functions, our data suggest a crosstalk between the autophagy pathway and MHCII-restricted Ag presentation in LECs impacting T reg cells. In addition, our results revealed a novel role of autophagy in LECs in regulating the degradation of kinases implicated in the production of S1P, therefore inhibiting pathogenic effector Th17 cell egress from LNs and dampening T cell–mediated autoimmunity. Altogether, our study brings new insight into the implication of autophagy in the immunomodulatory functions of LECs under inflammatory settings.

## Results

### LC3^+^ autophagosomes are induced in LECs during CIA

**Figure 1.**
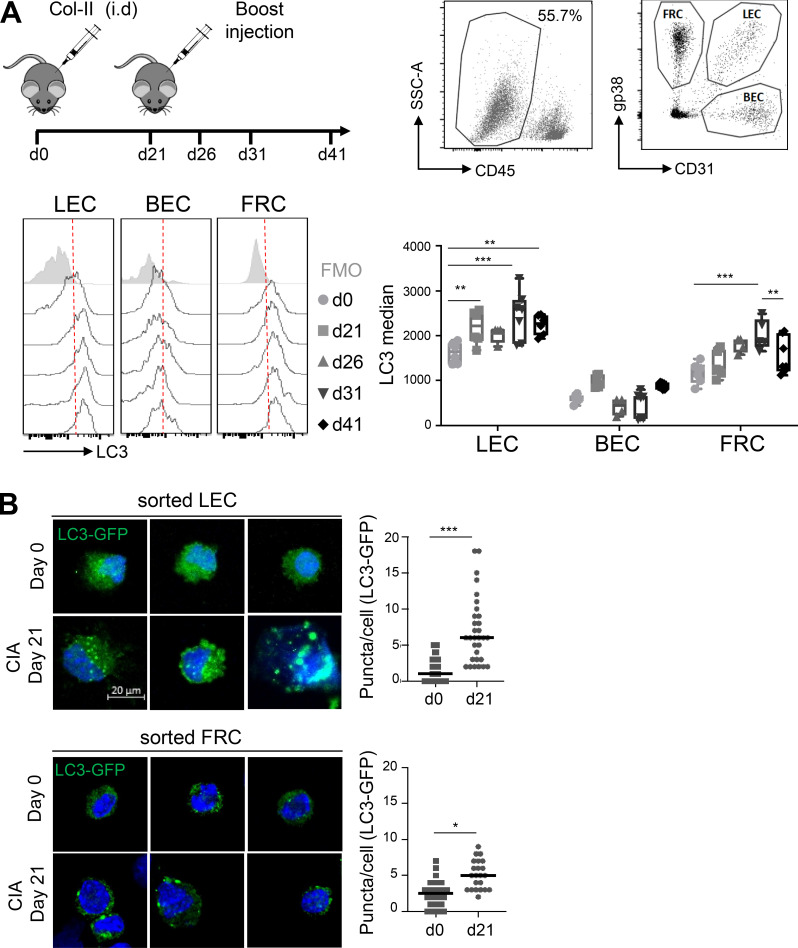
**CIA-mediated inflammation induces autophagy in LECs. (A and B)** CIA was induced in WT (A) and LC3-GFP (B) mice by injecting an emulsion of CFA with chicken type II collagen (col-II). **(A)** Intracellular LC3-II levels (LC3 median–FMO) were assessed by flow cytometry in LECs, BECs, and FRCs at indicated time points. Data are representative of two independent experiments with four or five mice for each time point. Error bars correspond to lower and higher values for each group. Two-way ANOVA. **(B)** LECs and FRCs were sorted from dLNs at day 0 and day 21, and autophagosomes (intracellular LC3-GFP^+^ organelles) were assessed by microscopy. Representative images are provided. Histograms represent the number of GFP^+^ organelles/cell. Data are pooled from five individual mice. Unpaired *t* test; * P < 0.05, ** P < 0.01, and *** P < 0.001.

To determine whether autophagy in LNSCs regulates autoimmunity, we first assessed intracellular LC3-II levels in LECs, BECs, and FRCs under autoimmune settings in vivo. We used the mouse model of rheumatoid arthritis, CIA. C57BL/6 mice were immunized at day 0 and day 21 (boost) with chicken type II collagen (col2a1; Inglis et al., 2008). Autophagosome formation was evaluated in LNSCs at different time points (Fig. 1 A). We used a fluorescently labeled antibody against LC3 combined with a permeabilization buffer, allowing the release of the LC3 cytoplasmic form (LC3-I) while the membrane form (LC3-II), associated with autophagosome membranes, is retained inside the cells. Steady-state LN LECs, BECs, and FRCs (day 0) exhibited detectable basal levels of intracellular LC3-II (Fig. 1 A), with BECs exhibiting lower levels compared with the other two populations. In addition, whereas BECs maintained negligible levels of intracellular LC3-II upon CIA development in LNs draining the site of immunization, both LECs and FRCs demonstrated a significant increase in intracellular LC3-II levels at different time points during CIA (Fig. 1 A). Interestingly, the kinetics of up-regulation appeared different in LECs and FRCs, with earlier induction of the pathway in LECs (from day 21, significant fold change of 1.4 related to day 0) compared with FRCs (from day 31, significant fold change of 1.7 related to day 0). These results were confirmed in LECs and FRCs sorted by flow cytometry from LNs draining the site of CIA induction in LC3-GFP mice (Mizushima et al., 2004). Both populations demonstrated low basal levels of intracellular LC3-GFP^+^ autophagosomes at steady state and a significant induction of LC3-GFP^+^ autophagosome formation at day 21 after CIA induction (Fig. 1 B). Again, the fold increase of LC3-GFP^+^ organelles upon CIA was higher in LECs compared with FRCs at day 21 (five vs. two, respectively), suggesting that LECs activate the autophagy pathway earlier compared with FRCs during CIA development.

### Autophagy in LECs regulates the development of CIA

**Figure 2.**
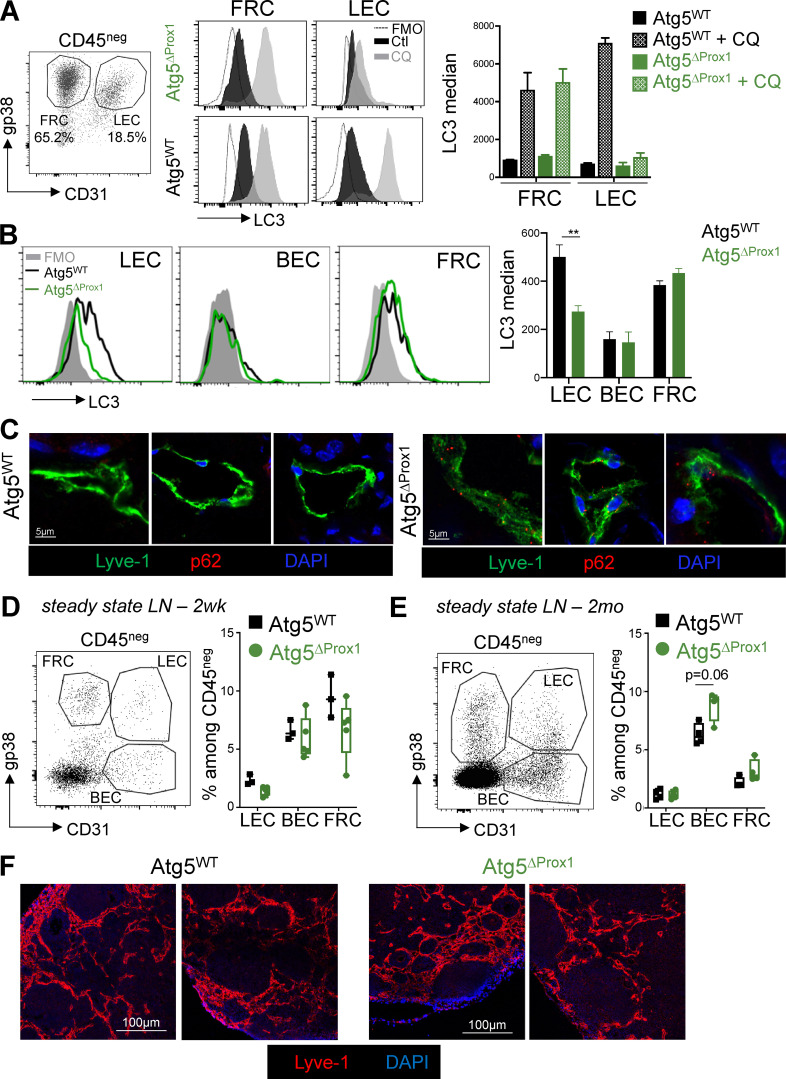
**Autophagy is abolished in LNs and skin LECs from Atg5^ΔProx1^ mice but does not impair stromal frequencies and lymphatic vessel organization in steady-state LNs.** **(A–F)** Atg5^WT^ and Atg5^ΔProx1^ mice were treated with Tx. **(A)** LEC/FRC cultures from LNs of Atg5^WT^ and Atg5^ΔProx1^ mice were treated or not treated with CQ for 4 h, and intracellular LC3 levels (LC3-II) were assessed by flow cytometry (two independent experiments with two or three mice/group). **(B)** Atg5^WT^ and Atg5^ΔProx1^ mice were injected with IFN-γ and CQ. CD45^neg^ cells were isolated from skin LNs 24 h after, and intracellular LC3 levels (LC3-II) were assessed in LECs, BECs, and FRCs by flow cytometry. Unpaired *t* test; ** P < 0.01, three to five mice/group. **(A and B)** Error bars correspond to SEM. **(C)** Sections showing intracellular p62 in LECs (Lyve1^+^) from back skin of Atg5^WT^ and Atg5^ΔProx1^ mice 5 d after OVA + CFA immunization. Representative images, three independent mice/group. **(D and E)** LEC, BEC, and FRC frequencies in skin LNs 2 wk (2wk, gated on CD45^neg^Ter119^neg^; D) and 2 mo (2mo, gated on CD45^neg^; E) after Tx treatment. Error bars correspond to lower and higher values for each group. **(F)** 2 mo later, lymphatic vessels (Lyve1^+^) were analyzed on sections of skin LNs from Atg5^WT^ and Atg5^ΔProx1^ mice 2 mo after Tx treatment. Images show LNs from individual mice.

**Figure S1.**
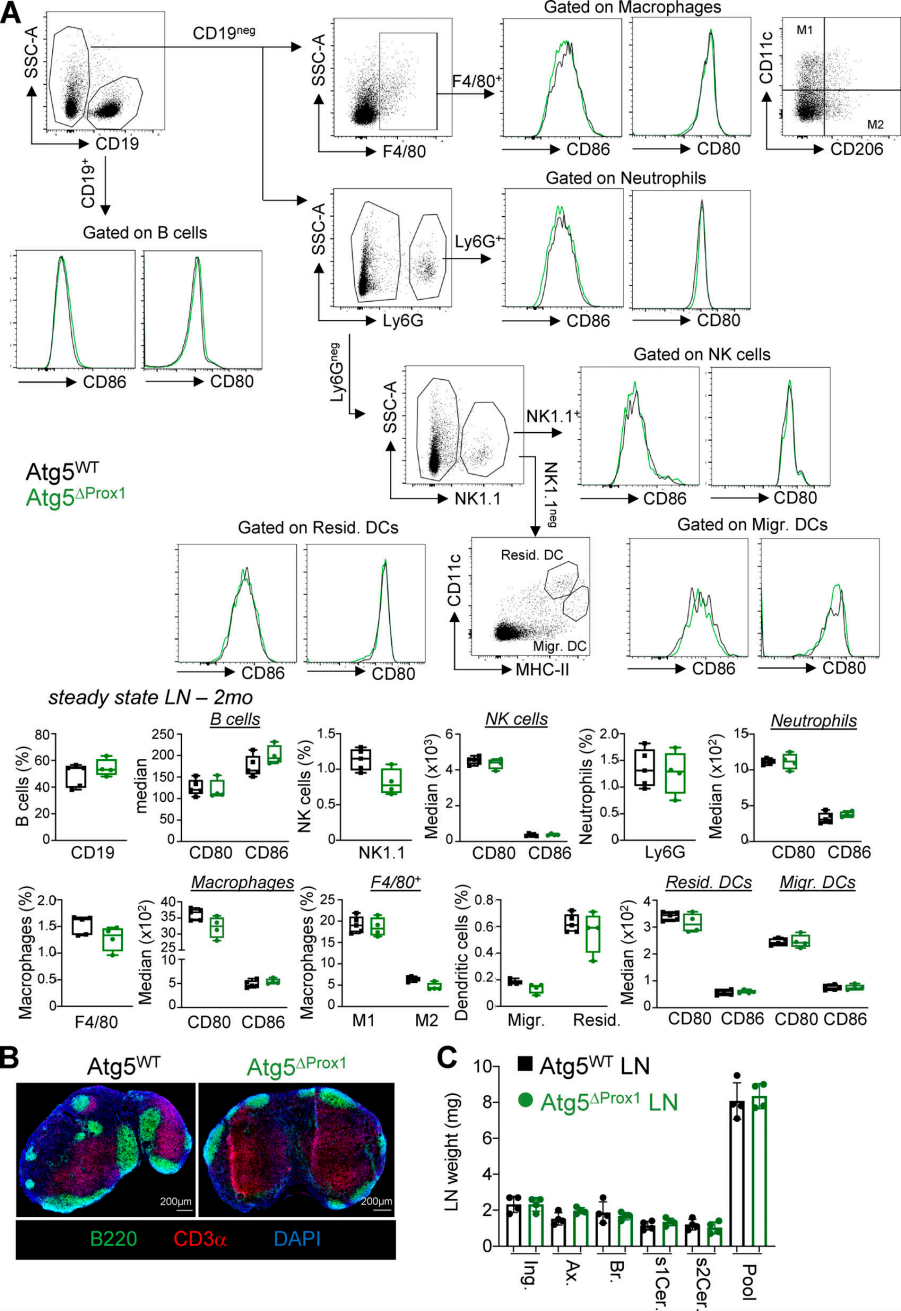
**Autophagy abrogation in LECs does not impair LN immune cell frequencies or LN organization at steady state.** Atg5^WT^ and Atg5^ΔProx1^ mice were treated with Tx. **(A)** B cells (CD19^+^), natural killer cells (NK1.1^+^), neutrophils (Ly6G^+^), macrophages (F4/80^+^), M1 (F4/80^+^CD11C^high^CD206^low^), M2 (F4/80^+^CD11clowCD206high macrophages, resident (CD11c^low^MHCII^high^), and migratory (CD11c^high^MHCII^low^) DC gating strategy and frequencies and expression levels of CD80 and CD86 in skin LNs 2 mo after Tx treatment. Unpaired *t* test, three or four mice/group. Error bars correspond to lower and higher values for each group. **(B)** T cell area (CD3^+^ cells) and B cell area (B220^+^ cells) in LNs 2 mo after Tx. Images are representative LNs from three independent mice/group. **(C)** Individual peripheral skin LN (Ing., inguinal; Ax., axial; Br., brachial; s1Cer. and s2Cer., superficial cervical 1 and 2; Pool) weights 2 mo after Tx treatment (four mice/group). Error bars correspond to SEM.

**Figure S2.**
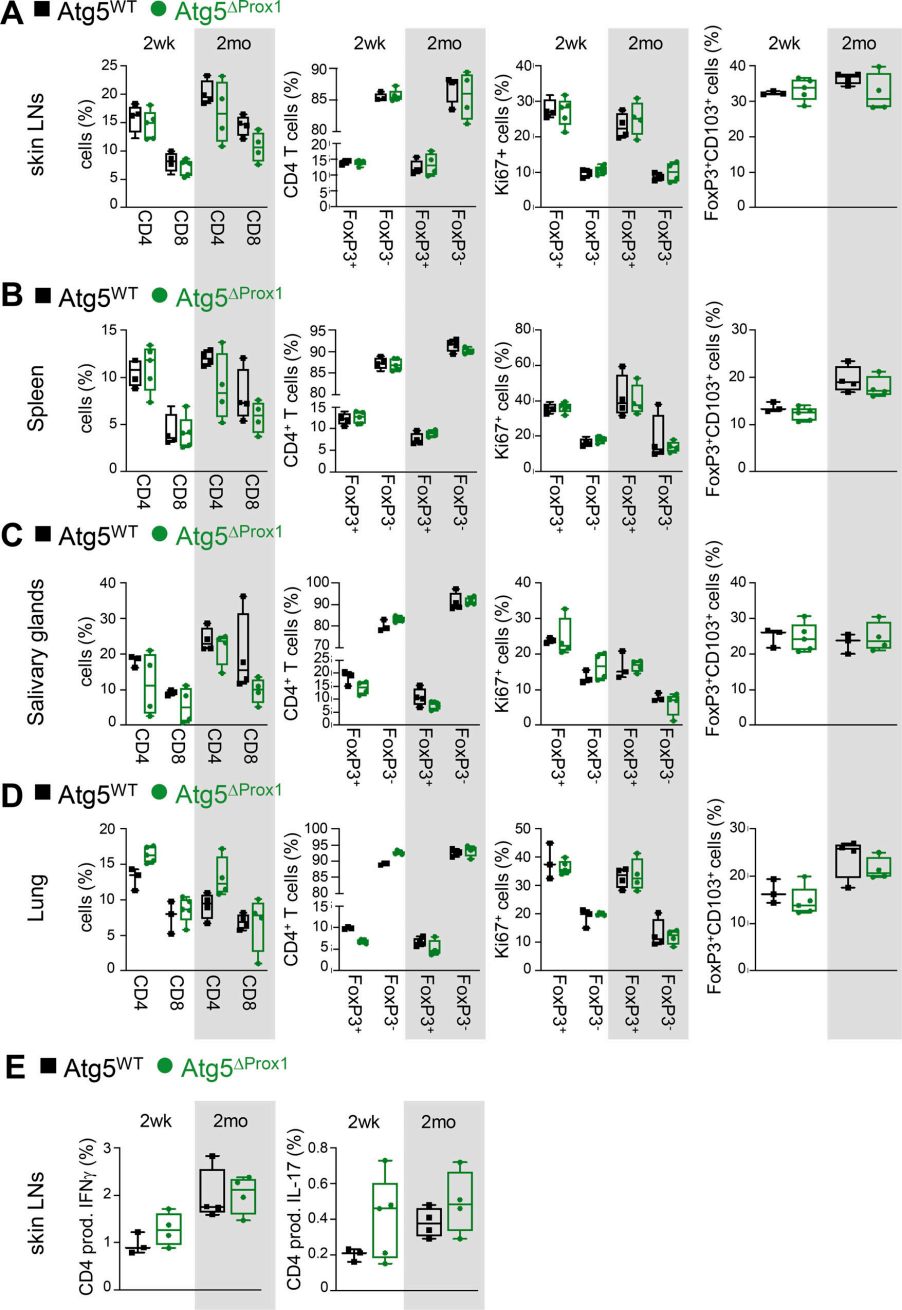
**Autophagy abolition in LECs does not impair LN organization and T cell homeostasis at steady state.** Atg5^WT^ and Atg5^ΔProx1^ mice were treated with Tx. **(A–D)** CD4^+^, CD8^+^ T cell, and T reg cell (FoxP3^+^) frequencies, proliferation (Ki67^+^), and expression of CD103 among T reg cells (CD4^+^FoxP3^+^) in skin LNs (A), spleen (B), salivary glands (C), and lungs (D) assessed by flow cytometry 2 wk and 2 mo after Tx treatment. **(E)** Frequencies of CD4^+^ T cells producing IFN-γ (Th1) and IL-17 (Th17) in LNs 2 wk (2wk) and 2 mo after Tx. Unpaired *t* test; three or four mice/group. **(A–E)** Error bars correspond to lower and higher values for each group.

To address whether the early and significant induction of autophagy in LECs during CIA has an impact on disease development, we generated genetically modified mice in which autophagy is deleted in LECs. Prox1cre^ERT2^ mice, which express the tamoxifen (Tx)-inducible cre recombinase under the promoter of the transcription factor Prox1 (Bazigou et al., 2011), essential for LEC differentiation and maintenance, were crossed with Atg5^flox^ mice. Atg5 is one of the autophagy-related proteins indispensable for the early steps of autophagosome formation (Ye et al., 2018; Hara et al., 2006). To verify the specific deletion of autophagosome formation in LECs in Prox1cre^ERT2^ × Atg5^flox^ (Atg5^ΔProx1^) mice upon Tx treatment, LECs and FRCs were generated from LN cultures of Atg5^ΔProx1^ and Atg5^flox^ (Atg5^WT^) mice as controls. Cells were cultured either in complete medium or in starvation condition (in HBSS devoid of FBS) and treated with chloroquine (CQ) to induce and stabilize autophagosomes. After starvation, a significant increase of LC3-II levels was observed in both LECs and FRCs from Atg5^WT^ mice, confirming the ability of LECs and FRCs to induce autophagy in vitro. In contrast, whereas FRCs from Atg5^ΔProx1^ mice retained this ability, the increase of LC3-II levels in LECs from Atg5^ΔProx1^ mice was fully abrogated, validating in vitro the abrogation of autophagosome formation in LECs in these mice (Fig. 2 A). To further validate the conditional deletion of autophagy in LECs in vivo, Atg5^ΔProx1^ and Atg5^WT^ mice were treated with IFN-γ to induce inflammation and CQ to accumulate the autophagosomes. LC3 staining demonstrated that LC3-II^+^ autophagosomes accumulated in LN LECs and FRCs from Atg5^WT^ mice, whereas BECs maintained very low levels of LC3, the median being comparable to the FMO (full minus one; Fig. 2 B). In contrast, whereas FRCs and BECs from Atg5^ΔProx1^ mice behaved as their control littermates, LN LECs from Atg5^ΔProx1^ mice exhibited a significant LC3 median reduction compared with Atg5^WT^ LECs (Fig. 2 B). Moreover, to determine whether autophagy was also deleted in skin LECs during inflammation, we measured the accumulation of the protein p62, which interacts with ubiquitinylated proteins to target them toward autophagosomes and is therefore itself degraded when the pathway is active (Lippai and Lőw, 2014). P62 was stained on back skin sections from Atg5^ΔProx1^ and Atg5^WT^ mice immunized with OVA + complete Freund’s adjuvant (CFA). We did not observe any p62 staining in skin LECs from immunized Atg5^WT^ mice, indicating that under inflammation, the protein is degraded by the autophagy pathway. In contrast, p62 accumulated in skin LECs from immunized Atg5^ΔProx1^ mice, confirming the abrogation of autophagy in these cells (Fig. 2 C). Therefore, autophagy is efficiently abrogated in LN and skin LECs from Atg5^ΔProx1^ mice. 2 wk and 2 mo after Tx treatment, naive Atg5^ΔProx1^ and Atg5^WT^ mice did not reveal any alteration in LNSC repartition in LNs (Fig. 2, D and E). Lyve-1 staining on Atg5^ΔProx1^ and Atg5^WT^ steady-state LN sections did not indicate any alterations of lymphatic vessel structure (Fig. 2 F). In addition, frequencies of B cells, macrophages, and LN resident and migratory DCs, as well as their expression of costimulatory molecules (CD80, CD86), were not altered in LNs from naive Atg5^ΔProx1^ and Atg5^WT^ mice 2 mo after Tx injection (Fig. S1 A). Similarly, we did not observe any effect on natural killer cell and neutrophil frequencies (Fig. S1 A). LN structure did not seem to be affected by the Tx treatment at steady state, exhibiting normal T and B cell zones (Fig. S1 B), and peripheral LN weights were found to be similar as well (Fig. S1 B). Accordingly, 2 wk and 2 mo after Tx treatment, naive Atg5^ΔProx1^ and Atg5^WT^ mice did not reveal any alteration in the T cell compartment, neither in lymphoid organs (such as skin LNs and spleen; Fig. S2, A and B) nor in peripheral tissues (such as in salivary glands and lungs; Fig. S2, C and D). Finally, the frequencies of Th1 and Th17 cells in LNs are also similar at steady state in Atg5^ΔProx1^ and Atg5^WT^ LNs (Fig. S2 E). Altogether, our results indicate that the immune system in Atg5^ΔProx1^ mice is similar to that of Atg5^WT^ mice at the baseline before CIA induction, indicating that autophagy in LECs does not impact naive T cell homeostasis at steady state.

**Figure 3.**
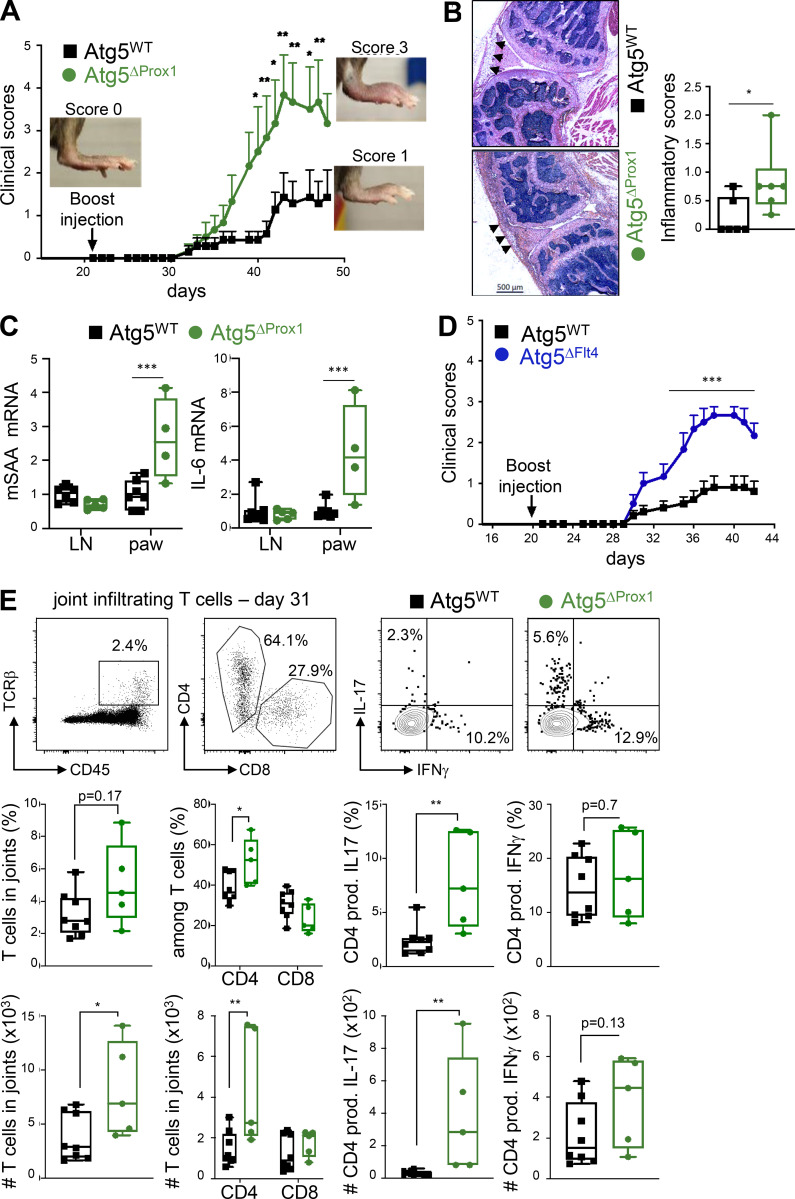
**Autophagy in LECs dampens CIA severity. (A–C)** CIA was induced in Tx-treated Atg5^WT^ and Atg5^ΔProx1^ mice. **(A)** Clinical scores (paw thickness) were assessed at indicated time points. Data are representative of two independent experiments with six to eight mice/group. **(B)** Inflammatory scores were assessed by histology analysis of knee sections at day 50 (six or seven mice/group). Representative images are provided, and arrowheads indicate inflammatory areas. Histograms represent global inflammation scores (sum of two knees per mouse). **(C)** mSAA and IL-6 mRNA levels in dLNs and paws at day 31 of CIA (four to six mice/group). **(D)** CIA was induced in Tx-treated Atg5^WT^ and Atg5^ΔFlt4^ mice. Clinical scores (paw thickness) were assessed at indicated time points. Data are representative of two independent experiments with six to eight mice/group. **(E)** CIA was induced in Tx-treated Atg5^WT^ and Atg5^ΔProx1^ mice. Flow cytometry analysis of indicated T cell subsets infiltrating hind-joint legs at day 31 (five or six mice/group). Representative dot plots are provided. Histograms represent the frequencies or the numbers of indicated cells in joints. Two-way ANOVA (A and D) and unpaired *t* test (B, C, and E). * P < 0.05, ** P < 0.01, and *** P < 0.001. Error bars correspond to lower and higher values for each group (B, C, and E) and to SEM (A and D).

We next assessed the impact of autophagy deletion in LECs during CIA by evaluating clinical scores (paw thickness) in Atg5^ΔProx1^ and Atg5^WT^ mice. Both groups exhibited similar disease onset (at day 31; Fig. 3 A). However, Atg5^ΔProx1^ mice developed more severe clinical scores compared with Atg5^WT^ mice (mean score of 4 at peak disease in Atg5^ΔProx1^ compared with 1.5 in Atg5^WT^ mice; Fig. 3 A) associated with an increased incidence (100% in Atg5^ΔProx1^ compared with 75% in Atg5^WT^ mice at day 48). Disease exacerbation was confirmed by histological analysis, which revealed increased inflammatory scores in knees from Atg5^ΔProx1^ compared with Atg5^WT^ mice (Fig. 3 B). Furthermore, mRNA levels of inflammatory markers described as playing key roles during the acute phase of CIA, such as IL-6 and serum amyloid A (SAA; Yoshida and Tanaka, 2014; Cunnane et al., 2000), were increased in paws from Atg5^ΔProx1^ mice at day 31 of CIA compared with Atg5^WT^ mice (Fig. 3 C). To confirm the role of autophagy in LECs on disease severity, CIA was induced in a second conditional LEC knockout expressing Tx-inducible cre recombinase under the promoter Flt4 (VEGF-C receptor, expressed by LECs) crossed with Atg5^flox^ mice (Atg5^ΔFlt4^). Similar to Atg5^ΔProx1^ mice, clinical scores were exacerbated and CIA incidence increased in Atg5^ΔFlt4^ compared with Atg5^WT^ mice (Fig. 3 D; and incidence of 100% in Atg5^ΔProx1^ compared with 60% in Atg5^WT^ mice at day 48).

We next analyzed T cells infiltrating the hind leg joints from Atg5^ΔProx1^ and Atg5^WT^ mice at day 31 after CIA. Infiltrating T cell frequencies and numbers, and mostly CD4^+^ T cells, were significantly increased in joints from Atg5^ΔProx1^ mice compared with Atg5^WT^ mice (Fig. 3 E). No variation was observed for Th1 cell populations. In contrast, effector Th17 cell frequencies and numbers were significantly augmented in joints from Atg5^ΔProx1^ mice (Fig. 3 E), which is consistent with the increased CIA severity observed in these mice (Fig. 3 A). Altogether, our data demonstrate that the abrogation of autophagy in LECs results in CIA exacerbation and enhanced Th17 cells in joints.

### Does autophagy contribute to MHCII-restricted Ag presentation in LECs?

**Figure 4.**
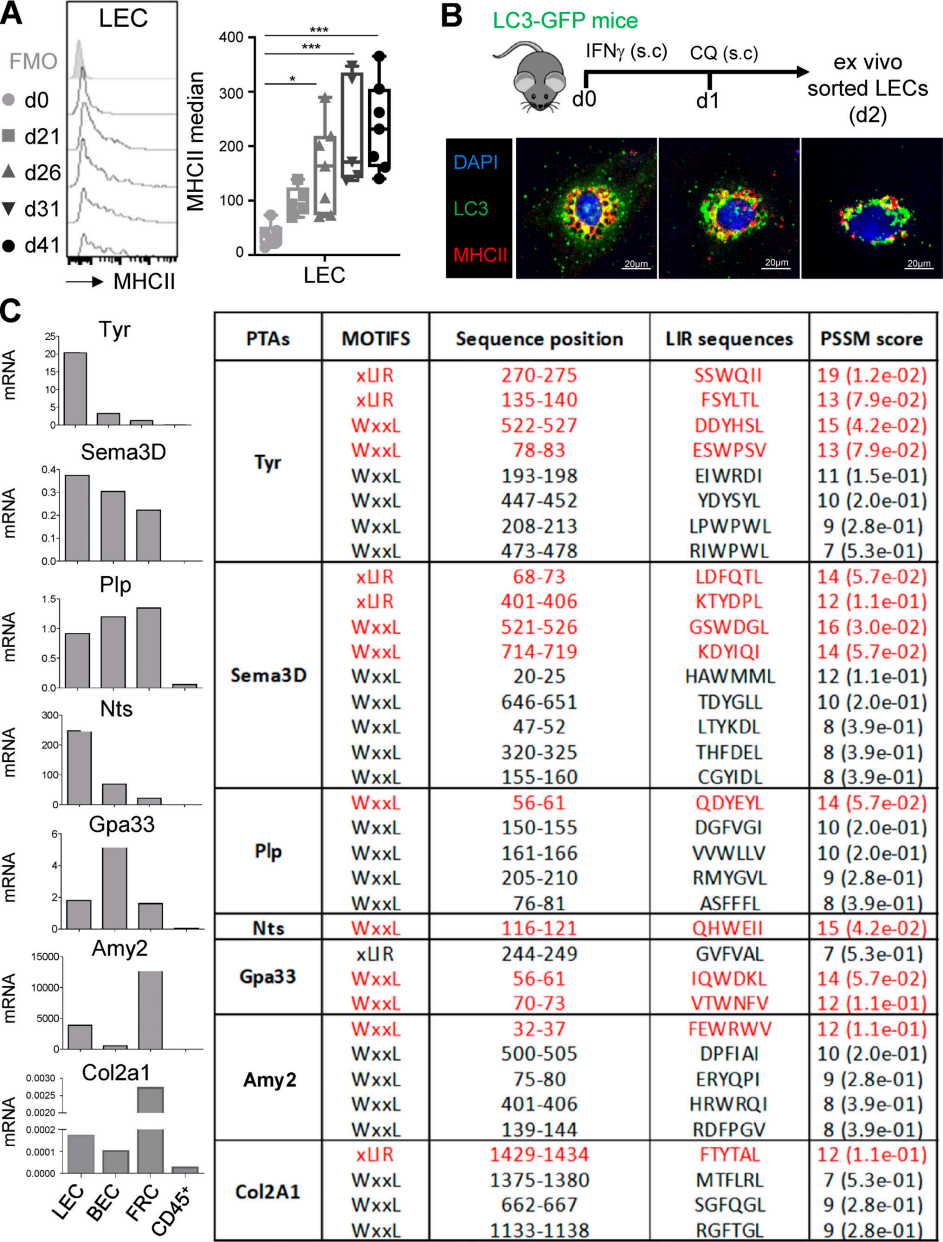
**Contribution of autophagy to MHCII-restricted presentation of PTA by LECs. (A)** CIA was induced in WT mice, and MHCII expression levels were assessed by flow cytometry in dLN LECs at indicated time points. Data represent MHCII expression levels (median–FMO) and are representative of two independent experiments with four or five mice for each time point. One-way ANOVA; * P < 0.05 and *** P < 0.001. Error bars correspond to lower and higher values for each group. **(B)** LC3-GFP mice were injected s.c. with IFN-γ and CQ. LECs were sorted from dLNs, and colocalization of autophagosomes (LC3-GFP^+^ organelles) and MHCII compartment was examined by microscopy. **(C)** Pattern of indicated PTA mRNA expressed by LECs, BECs, and FRCs sorted from steady-state LNs (pool of three or four mice) and PSSM scores of their amino acid sequences determining their potential targeting toward autophagosomes (iLIR database software).

It was previously demonstrated that autophagosomes can target some Ags to MHCII^+^ compartments in mTECs and DCs (Aichinger et al., 2013; Münz, 2009). Therefore, we wondered whether autophagy abolition in LECs could impact their MHCII-restricted Ag-presenting functions and affect CD4^+^ T cell responses. We first assessed MHCII levels in LECs during CIA. LECs significantly up-regulated MHCII molecules as early as 26 d after CIA induction (4.3-fold change compared with day 0; Fig. 4 A). Interestingly, MHCII up-regulation and intracellular LC3-II accumulation in LECs during CIA (Fig. 1) followed similar kinetics. To determine whether a crosstalk exists between autophagy and MHCII-restricted Ag presentation pathways in LECs, LC3-GFP mice were treated with IFN-γ to induce MHCII up-regulation in LECs and further treated with CQ to stabilize autophagosomes. LECs sorted from LNs draining the immunization site by flow cytometry 24 h later demonstrated an intracellular colocalization of MHCII^+^ organelles with LC3-GFP^+^ autophagosomes, suggesting an efficient targeting of autophagosomes to MHCII loading compartments (Fig. 4 B). LNSCs express a broad range of PTAs (Cohen et al., 2010; Fletcher et al., 2010; Gardner et al., 2008; Lee et al., 2007; Nichols et al., 2007; Yip et al., 2009). Interestingly, the type II collagen (col2a1), the endogenous protein used to induce CIA in mice, was described as being expressed by LECs, BECs, and FRCs (Malhotra et al., 2012). We confirmed the expression of some PTA mRNAs in LNSCs, including the col2a1 (Fig. 4 C), and determined whether some amino acid sequences in LNSC-expressed PTAs would predict their targeting to autophagosomes. Most of the PTAs tested contained amino acid sequences matching with LC3-interacting (LIR) motifs in the iLIR server (Kalvari et al., 2014) and predicting an efficient targeting to the autophagy pathway (Fig. 4 C). Position-specific scoring matrix (PSSM) scores of >12 were shown to give reliable predictions for an attachment to LC3 (Klionsky et al., 2016). Therefore, PTAs expressed by LNSCs exhibit the potential to be targeted toward autophagosomes. Together with a substantial colocalization between MHCII^+^ organelles and LC3^+^ compartments in LECs, our results indicate a possible crosstalk between autophagy and MHCII-restricted Ag presentation pathway in these cells.

### MHCII abrogation in LECs exacerbates CIA

**Figure 5.**
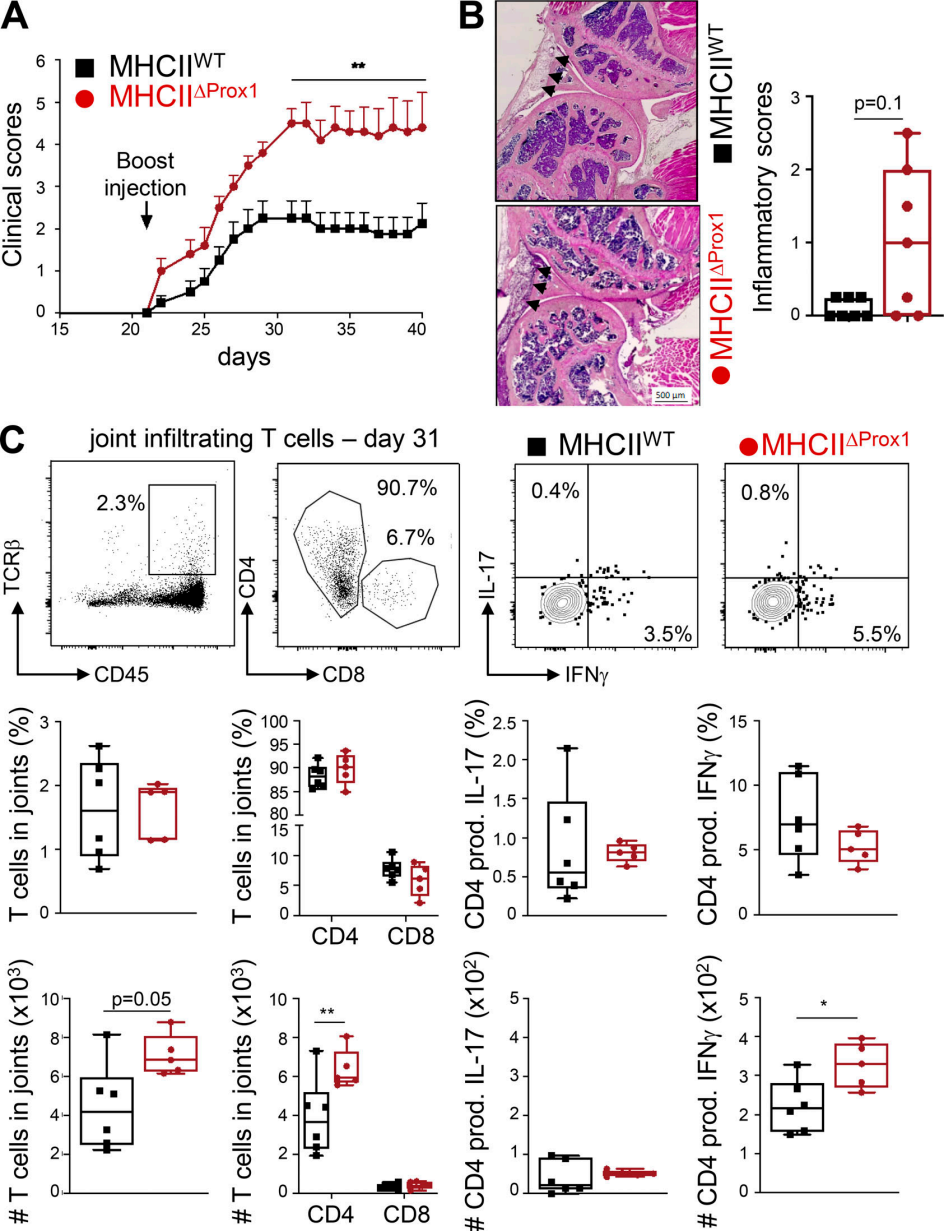
**Abolition of MHCII in LECs exacerbates CIA.** **(A–C)** CIA was induced in Tx-treated MHCII^WT^ and MHCII^ΔProx1^ mice. **(A)** Clinical scores (paw thickness) were assessed at indicated time points. Data are representative of two independent experiments with six to eight mice/group. **(B)** Inflammatory scores were assessed by histological analysis on knee sections at day 41 (seven or eight mice/group). Representative images are provided, and arrowheads indicate areas of inflammation. Histograms represent global inflammation scores (sum of two knees per mouse). **(C)** Flow cytometry analysis of indicated T cell subsets infiltrating hind-joint legs at day 31, five or six mice/group. Representative dot plots are provided. Histograms represent the frequencies or the numbers of indicated cells in joints. Two-way ANOVA (A) and unpaired *t* test (B and C); * P < 0.05 and ** P < 0.01. Error bars correspond to SEM (A) and to lower and higher values for each group (B and C).

We next induced CIA in mice lacking MHCII expression by LECs generated through the crossing of Prox1cre^ERT2^ mice with MHCII^flox^ (MHCII^ΔProx1^) mice. Similar to Atg5^ΔProx1^ mice, MHCII^ΔProx1^ mice exhibited an increased disease severity compared with MHCII^flox^ (MHCII^WT^) control mice, as demonstrated by enhanced clinical scores (Fig. 5 A). Moreover, histological analysis of MHCII^ΔProx1^ knees revealed a tendency of enhanced inflammation compared with MHCII^WT^ control knees (Fig. 5 B). Although T cell frequencies were unaffected, numbers of T cells, mainly CD4^+^ T cells, infiltrating the hind leg joints were increased in MHCII^ΔProx1^ mice 31 d after CIA immunization (Fig. 5 C). In contrast to Atg5^ΔProx1^ mice, Th17 remained unchanged in joints from CIA MHCII^ΔProx1^ mice compared with controls. However, infiltrated Th1 cell numbers were significantly increased in joints from MHCII^ΔProx1^ mice (Fig. 5 C), supporting CIA exacerbation in these animals.

### Abolition of autophagy or MHCII in LECs results in T reg alterations in draining LNs (dLNs) during CIA

**Figure 6.**
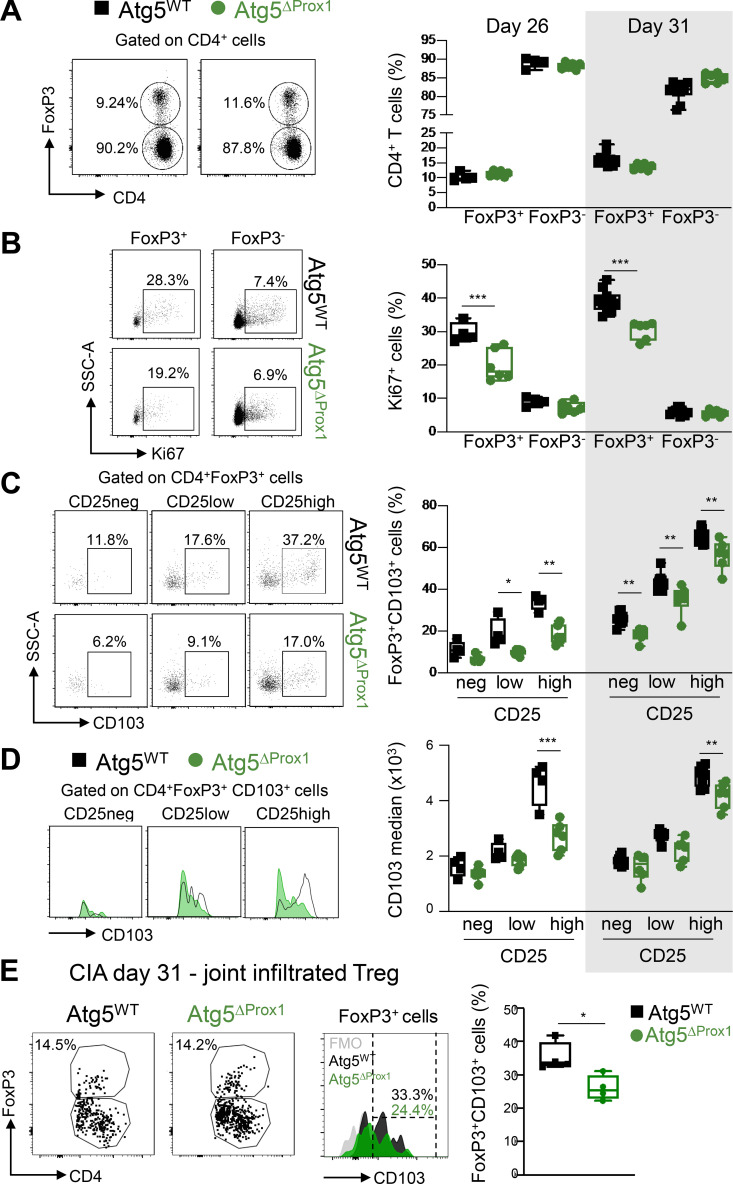
**Abolition of autophagy in LECs alters LN T reg cells during CIA. (A–E)** CIA was induced in Tx-treated Atg5^WT^ and Atg5^ΔProx1^ mice. **(A and B)** Flow cytometry analysis of T reg cells (FoxP3^+^CD4^+^ cells) and T conv cells (FoxP3^−^CD4^+^ cells) in dLNs at days 26 and 31. Cell frequency (A) and proliferation rate (Ki67^+^; B) in the CD4^+^ T cell population. **(C and D)** CD103 frequencies (C) and median (D) among T reg cells (CD4^+^FoxP3^+^; C) and CD103^+^ T reg cells (CD4^+^FoxP3^+^CD103^+^; D), CD25 negative (neg), or expressing low or high levels of CD25 in dLNs at days 26 and 31. Data are representative of two experiments with five or six mice/group each. **(E)** FoxP3^+^ frequency among CD4^+^ T cells and CD103 expression levels (median) among FoxP3^+^CD4^+^ T cells at day 31. Data are from one experiment with four or five mice/group. Unpaired *t* test (A and B) and two-way ANOVA (C and D). * P < 0.05, ** P < 0.01, and *** P < 0.001. **(A–E)** Error bars correspond to lower and higher values for each group.

**Figure S3.**
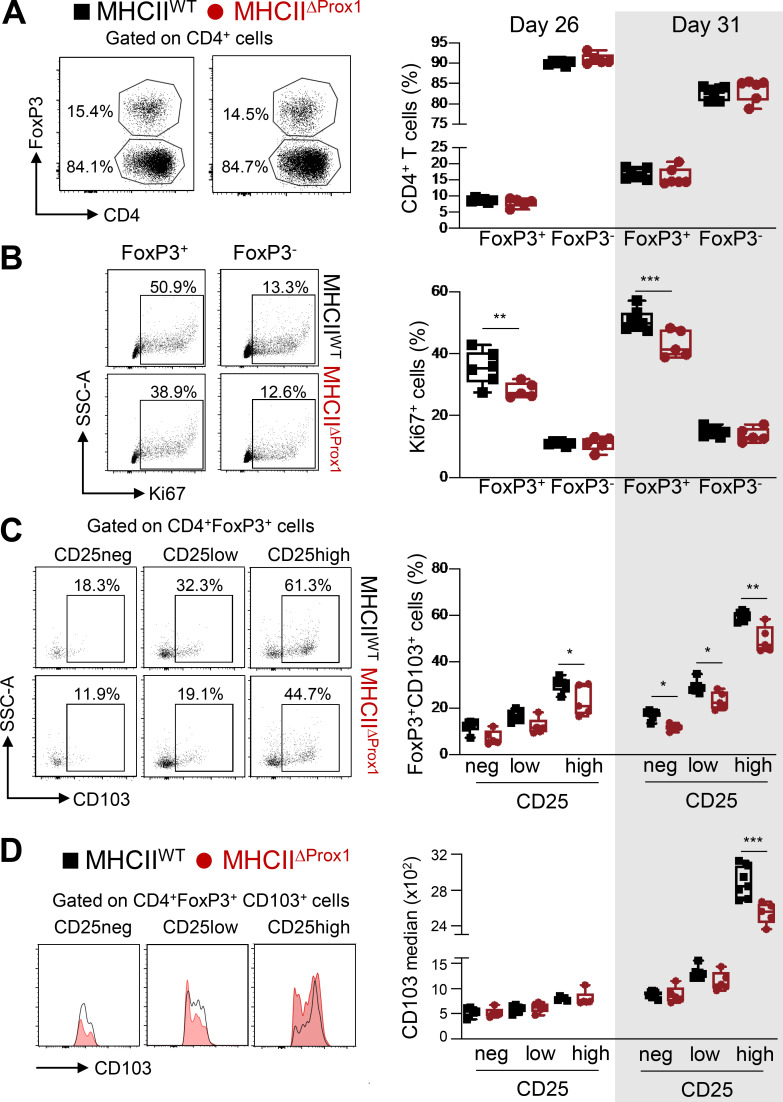
**Abolition of MHCII in LECs impacts T reg cells during CIA.** CIA was induced in Tx-treated MHCII^WT^ and MHCII^ΔProx1^ mice, and T reg cells were analyzed in dLNs at days 26 and 31. **(A and B)** CD4^+^Foxp3^+^ cells (T reg cells) and T conv (CD4^+^Foxp3^−^) cell frequencies (A) and proliferation rate (Ki67^+^; B). **(C and D)** CD103 frequencies (C) and median (D) among T reg cells (CD4^+^FoxP3^+^; C) and CD103^+^ T reg cells (CD4^+^FoxP3^+^CD103^+^; D), CD25 negative (neg), or expressing low or high levels of CD25. Five or six mice/group, two independent experiments. Unpaired *t* test (A and B) and two-way ANOVA (C and D). * P < 0.05, ** P < 0.01, and *** P < 0.001. **(A–D)** Error bars correspond to lower and higher values for each group.

We and others have previously shown that MHCII expression in LECs is important for T reg cell programming (Dubrot et al., 2018; Nadafi et al., 2020). At earlier time points (days 26 and 31 after CIA induction), although no variation in T reg cell (CD4^+^FoxP3^+^) frequency was observed in Atg5^ΔProx1^ and MHCII^ΔProx1^ dLNs (Fig. 6 A and Fig. S3 A), T reg cells were found to be less proliferative (Ki67^+^) than their respective control mice, while the proliferation of conventional T cell (T conv cell; CD4^+^FoxP3^−^) was not affected (Fig. 6 B and Fig. S3 B). T reg cells can be further separated based on their expression levels of CD25 (neg, low, and high; Fig. 6, C and D; and Fig. S3, C and D), the highest expression having been correlated with enhanced T reg cell suppressive functions (Miyao et al., 2012; Komatsu et al., 2009).

CD103 is an α-E integrin described as playing an important role in T reg cell retention at inflamed sites (Suffia et al., 2005). We observed that CD103 expression levels by T reg cells positively correlated to their expression levels of CD25 (Fig. 6, C and D; and Fig. S3, C and D). However, the frequency of T reg cells expressing CD103, as well as CD103 levels of expression by LN T reg cells from Atg5^ΔProx1^ and MHCII^ΔProx1^ mice, was reduced compared with control mice (Fig. 6, C and D; and Fig. S3, C and D). Elevated CD103 expression was particularly impaired in the CD25^high^ T reg cell population in LNs from Atg5^ΔProx1^ and MHCII^ΔProx1^ mice (Fig. 6, C and D; and Fig. S3, C and D). In contrast, neither MHCII abrogation nor autophagy deletion in LECs alters the expression of PD1 and ICOS by T reg cells (not shown). Therefore, autophagy or MHCII abolition in LECs results in alteration in T reg cell proliferation and CD103 expression in dLNs. Both autophagy and MHCII deletion in LECs induced an increase in CIA severity, associated with decreased T reg cell frequencies and an altered T reg phenotype in dLNs. Interestingly, decreased expression of CD103 by LN T reg cells correlated with diminished levels of CD103 expression by T reg cells present in arthritic joints of Atg5^ΔProx1^ mice compared with control mice (Fig. 6 E).

### MHCII deletion, but not autophagy, in LECs impaired T reg cell suppressive functions under inflammatory conditions

**Figure 7.**
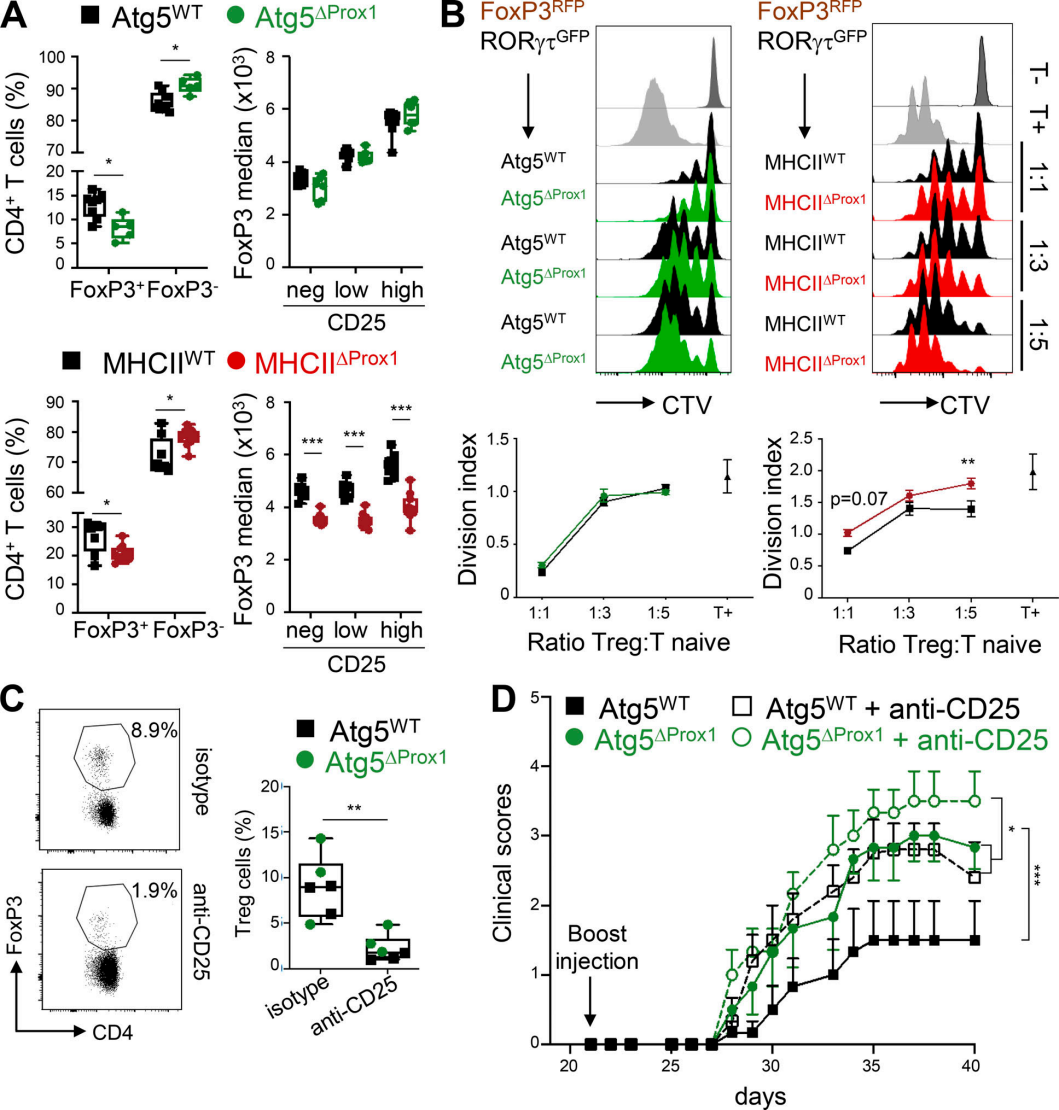
**Abolition of MHCII but not autophagy in LECs impairs intrinsic T reg cell suppressive functions during CIA. (A)** CIA was induced in Tx-treated MHCII^WT^ and MHCII^ΔProx1^ or Atg5^WT^ and Atg5^ΔProx1^ mice. FoxP3^+^ and FoxP3^−^ frequencies among CD4^+^ T cells as well as FoxP3 expression levels (median) among T reg cells (CD4^+^FoxP3^+^), CD25 negative (neg), or T reg cells expressing low or high levels of CD25 were assessed in dLNs from Tx-treated Atg5^WT^ and Atg5^ΔProx1^ mice and MHCII^WT^ and MHCII^ΔProx1^ mice at late time point after CIA induction (day 50 and day 41, respectively). **(B)** CIA was induced in Tx-treated FoxP3^RFP^RORγt^GFP^→Atg5^ΔProx1^, Atg5^WT^, MHCII^ΔProx1^, and MHCII^WT^ BM-chimeric mice. T reg cells (FoxP3^RFP^) were sorted at day 26 and co-cultured at indicated ratio with CTV-labeled CD4^+^CD25^neg^ cells stimulated with anti-CD3 antibodies and BM-derived DCs. After 3 d, the division index of CTV-labeled CD4^+^CD25^neg^ cells was analyzed by flow cytometry. **(A and B)** Data are representative of two independents experiments, six or seven mice/group. **(C and D)** CIA was induced in Tx-treated Atg5^WT^ and Atg5^ΔProx1^ mice and treated or not treated with depleting anti-CD25 antibodies. **(C)** CD4^+^FoxP3^+^ cell depletion was assessed in blood by flow cytometry at day 25. **(D)** Clinical scores (paw thickness) were assessed at indicated time points, six mice/group. Multiple *t* test comparison corrected using Sidak-Bonferroni method for T reg cell frequencies and two-way ANOVA for median of FoxP3 (A), two-way ANOVA (B and D), and unpaired *t* test (C). * P < 0.05, ** P < 0.01, and *** P < 0.001. Error bars correspond to lower and higher values for each group (A and C) and to SEM (B and D).

FoxP3 expression levels were positively correlated with T reg cell suppressive functions (Fontenot et al., 2003). Therefore, we characterized T reg cell frequencies and Foxp3 expression 20 d after CIA onset in LNs draining the immunization site of Atg5^ΔProx1^, MHCII^ΔProx1^, and their respective control mice. FoxP3^+^ T reg cell frequencies were decreased during the CIA chronical phase in both Atg5^ΔProx1^ and MHCII^ΔProx1^ mice compared with control animals (day 50 and day 41, respectively), associated with increased in T conv frequencies (Fig. 7 A). A positive correlation has been established between CD25 expression levels and an increased stability of the transcription factor FoxP3, together with enhanced suppressive functions (Komatsu et al., 2009; Miyao et al., 2012). FoxP3 expression was down-regulated in T reg cells from MHCII^ΔProx1^ dLNs, regardless of their expression levels of CD25 (Fig. 7 A), whereas FoxP3 expression levels remained unchanged in dLNs of Atg5^ΔProx1^ mice (Fig. 7 A). These results indicate that LN T reg cells might exhibit an altered ability to inhibit T cell activation in the absence of MHCII expression by LECs.

To further characterize whether MHCII expression and/or autophagy in LECs might regulate T reg cell suppressive functions during CIA, we performed ex vivo suppressive assays to assess the ability of T reg cells isolated from LNs of Atg5^ΔProx1^ and MHCII^ΔProx1^ CIA mice to inhibit T cell proliferation. To sort T reg cells based on a specific FoxP3 fluorescent reporter, we generated bone marrow (BM) chimeric mice. Lethally irradiated Atg5^ΔProx1^ and MHCII^ΔProx1^ mice and their respective controls were injected with BM cells from FoxP3-RFP mice. T reg cells (RFP^+^ cells) were sorted from dLNs of BM chimeras 26 d after CIA induction and cultured at different ratios with naive cell trace violet (CTV)–labeled CD25^−^CD4^+^ T cells stimulated with anti-CD3 antibodies and BM-derived DCs (Fig. 7 B). T reg cells from MHCII^ΔProx1^ mice exhibited an impaired ability to suppress the proliferation of naive T cells, as revealed by a higher division index (ratio 1:5) compared with naive T cells incubated with MHCII^WT^ T reg cells (Fig. 7 B). In contrast, T reg cells from Atg5^ΔProx1^ mice displayed suppressive functions comparable to T reg cells from control mice (Fig. 7 B). Therefore, although autophagy might contribute to MHCII-restricted Ag presentation by LECs, other unrelated pathways confer the ability of LECs to present Ags through MHCII molecules to promote T reg cell suppression.

To further decipher the contribution of T reg cells to CIA exacerbation in the absence of autophagy in LECs, Atg5^ΔProx1^ and Atg5^WT^ CIA mice were treated with an anti-CD25–depleting antibody. T reg cells were efficiently depleted in the blood of treated mice compared with untreated controls (Fig. 7 C). As expected, untreated Atg5^ΔProx1^ mice exhibited higher clinical scores compared with Atg5^WT^ mice (Fig. 7 D). Moreover, T reg cell depletion resulted in CIA exacerbation in Atg5^WT^ mice, confirming an implication of T reg cells in regulating disease development (Sun et al., 2018). Interestingly, T reg–depleted Atg5^WT^ mice still exhibited significantly lower clinical scores compared with T reg–depleted Atg5^ΔProx1^ mice (Fig. 7 D). Disease exacerbation between T reg–deleted Atg5^ΔProx1^ and Atg5^WT^ mice was less pronounced compared with the exacerbation observed in untreated mice, which can be explained by T reg cell alterations existing in the absence of autophagy LECs. However, under these settings of a slowly evolving inflammatory reaction, it is difficult to determine whether T reg cell alterations in Atg5^ΔProx1^ mice are a cause or a consequence of disease exacerbation. Altogether, our results reveal that alterations of the T reg cell compartment in the absence of autophagy in LECs do not entirely explain CIA exacerbation and that autophagy in LECs regulates other pathways implicated in the modulation of disease severity.

### Abolition of autophagy in LECs affects Th17 egress from LNs in an S1P-dependent manner

**Figure 8.**
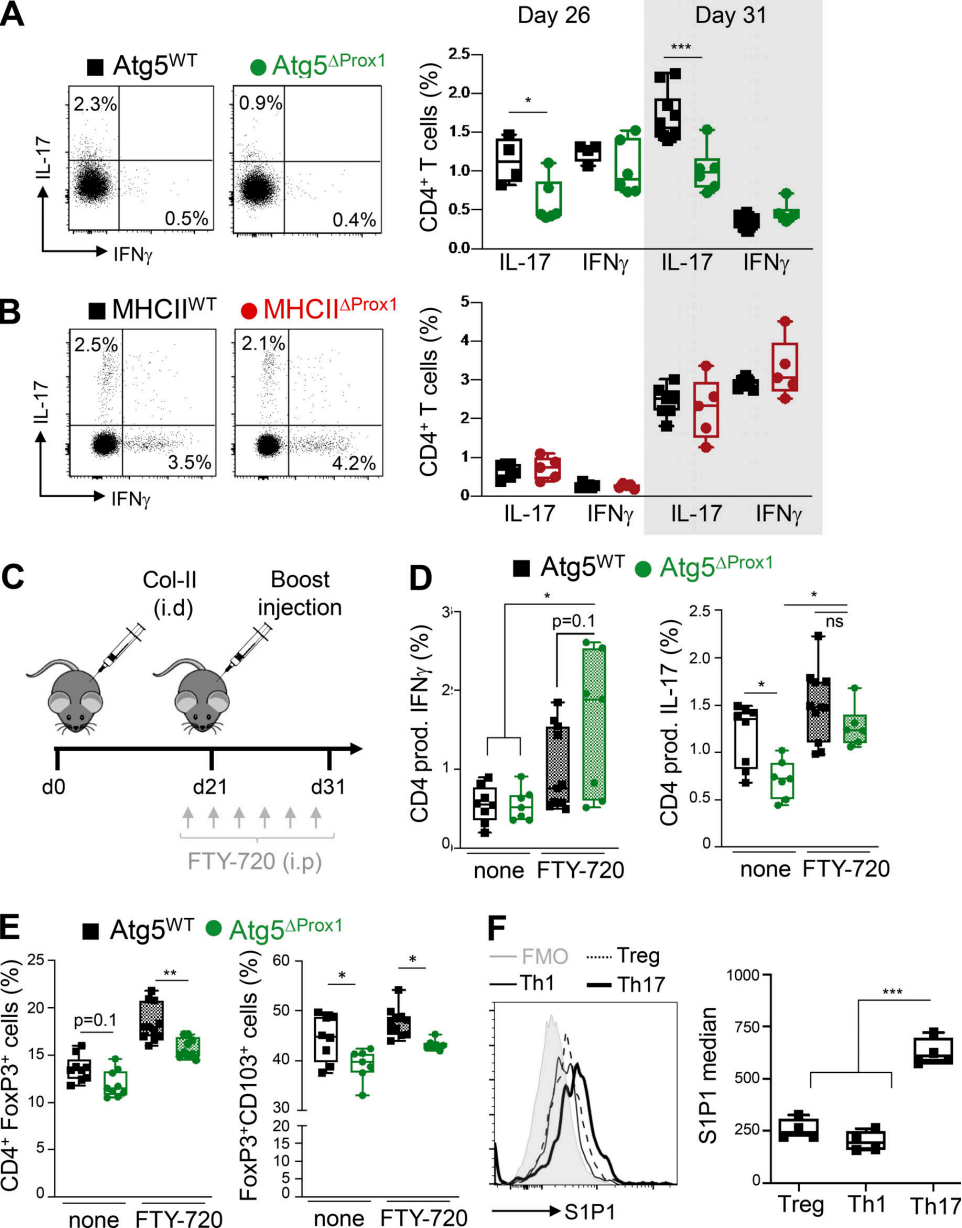
**Abolition of autophagy in LECs alters Th17 in dLNs during early phases of CIA. (A and B)** CIA was induced in Tx-treated Atg5^WT^ and Atg5^ΔProx1^ mice (A) and Tx-treated MHCII^WT^ and MHCII^ΔProx1^ mice (B), and IFN-γ–producing CD4^+^ T cell (Th1) and IL-17–producing CD4^+^ T cell (Th17) frequencies were analyzed in dLNs at indicated time points. **(C–E)** Tx-treated Atg5^WT^ and Atg5^ΔProx1^ CIA mice were injected with FTY-720 (C). **(D and E)** At day 31, IFN-γ–producing (Th1) and IL-17–producing (Th17; D), total T reg (FoxP3^+^) and CD103^+^ T reg (CD103^+^Foxp3^+^; E) CD4^+^ T cell frequencies were analyzed in dLNs by flow cytometry. Data are representative of two independent experiments with five to seven mice/group. **(F)** Expression levels of type 1 receptor of S1P (S1P1 median) were assessed by flow cytometry on T reg (FoxP3^+^), Th1 (IFN-γ^+^), and Th17 (IL-17^+^) CD4^+^ cells from dLNs of CIA WT mice at day 31. Data are representative of two independent experiments with four or five mice/group. Unpaired *t* test (A and B), multiple *t* test comparison corrected using the Sidak-Bonferroni method (D and E), and one-way ANOVA (F). * P < 0.05, ** P < 0.01, and *** P < 0.001. **(A–F)** Error bars correspond to lower and higher values for each group.

To decipher the additional mechanisms accounting for CIA exacerbation in Atg5^ΔProx1^ mice, we next characterized pathogenic Th1 and Th17 cells in LNs draining the site of disease induction at days 26 and 31. Whereas IFN-γ–producing Th1 CD4^+^ cell frequencies were not affected by the loss of autophagy in LECs, LNs from Atg5^Prox1^ mice surprisingly exhibited a significant decrease in the frequency of IL-17–producing Th17 CD4^+^ T cells compared with Atg5^WT^ mice (Fig. 8 A). This result was not observed in MHCII^ΔProx1^ mice (Fig. 8 B), supporting an MHCII-independent regulation of Th17 cell responses in mice lacking autophagy in LECs. The decrease of Th17 cells observed in LNs, together with the increase of joint-infiltrating Th17 cells (Fig. 3 E), might indicate a modulation of the Th17 organ migration pattern in Atg5^Prox1^ mice.

We hypothesized that autophagy in LECs regulates the homing of Th17 cells from LNs to inflamed joints during CIA. An important pathway by which LECs influence T cell responses is by regulating effector T cell egress from the LNs through the production of S1P. To determine whether the decrease of Th17 in dLNs of Atg5^ΔProx1^ is indeed a consequence of enhanced egress, CIA-induced Atg5^ΔProx1^ and Atg5^WT^ mice were treated or not with the S1P receptor antagonist FTY-720, which blocks lymphocyte egress from LNs (Fig. 8 C). In untreated animals, we recapitulated a significant decrease in Th17 frequencies at day 31 in dLNs from Atg5^ΔProx1^ compared with Atg5^WT^ mice, with again no variation in Th1 cells (Fig. 8 D). However, FTY-720–treated Atg5^ΔProx1^ and Atg5^WT^ mice exhibited similar frequencies compared with untreated mice for both Th1 and Th17 cells in dLNs (Fig. 8 D). These results demonstrate that autophagy in LECs does not impact the priming of pathogenic Th1 and Th17 cells during CIA, but rather, as also supported by the increase in Th17 cells in joints of arthritic Atg5^ΔProx1^ mice (Fig. 3 E), regulates the egress of Th17 cells from LNs. In striking contrast, decreased frequencies of both FoxP3^+^ T reg and of CD103^+^FoxP3^+^ T reg cells were maintained in LNs of Atg5^ΔProx1^ mice upon FTY-720 blockade, showing that the lack of autophagy in LECs alters the priming of CD103^+^ T reg cells in LNs upon CIA induction (Fig. 8 E). Therefore, although the pathway has no effect on the priming of pathogenic Th1 and Th17 cells, autophagy in LECs promotes the differentiation/expansion of CD103^+^ T reg cells in LNs, resulting in higher expression of CD103 by T reg cells localized in arthritic joints (Fig. 6 E).

Interestingly, LN exit of Th17 cells but not Th1 cells was impacted by the abrogation of autophagy in LECs, suggesting that different CD4^+^ T cell subsets might exhibit distinct levels of sensitivity to S1P gradients. Since the type 1 receptor for S1P, S1P1, is the main S1P receptor implicated in T cell egress (Garris et al., 2014), we assessed S1P1 expression levels in different CD4^+^ T cell subsets from LNs draining immunization sites day 31 after CIA induction. Strikingly, Th17 cells expressed significantly greater S1P1 levels compared with both T reg and Th1 cells (Fig. 8 F), which is consistent with the hypothesis that this particular effector T cell subset would be more sensitive to S1P gradients and their modulations by the autophagy pathway in LECs.

### Inflammation-induced autophagy modulates S1P production by regulating SphK1 levels in LECs to inhibit Th17 egress from LNs

**Figure 9.**
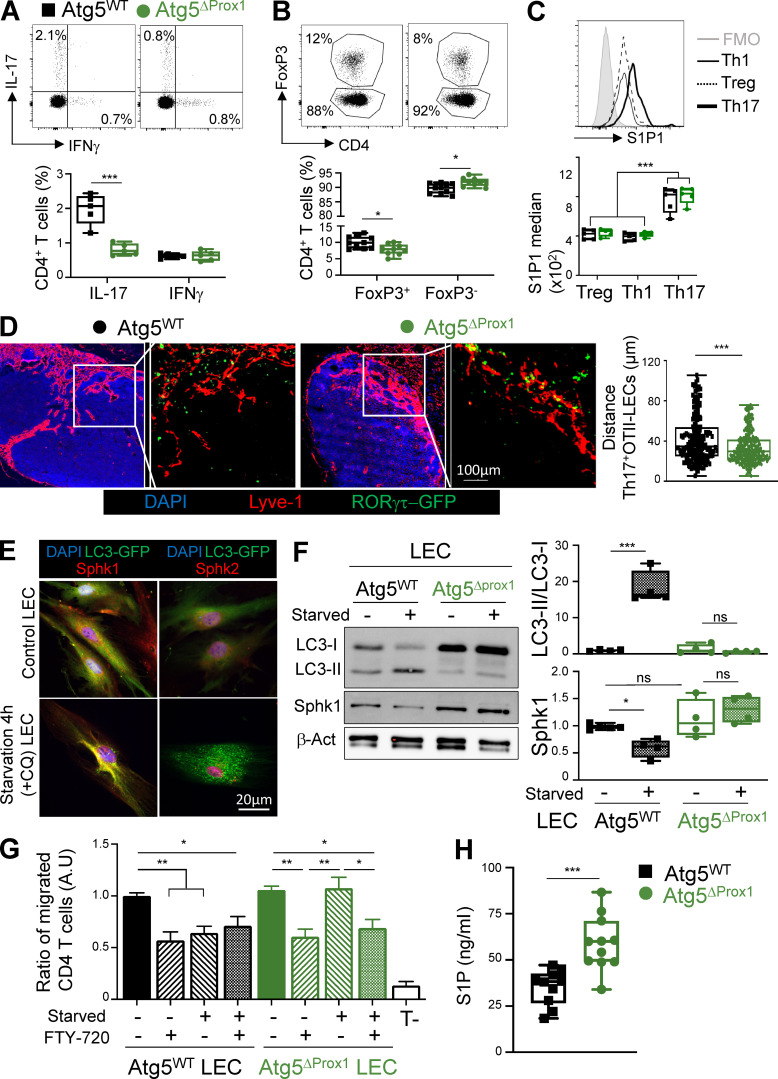
**Autophagy controls LEC ability to regulate effector T cell migration by inducing SphK1 degradation and decreasing S1P production. (A–C)** Tx-treated Atg5^WT^ and Atg5^ΔProx1^ mice were immunized with OVA_II_/CFA. 5 d later, IFN-γ–producing (Th1), IL-17–producing (Th17) CD4^+^ T cell frequencies (A), Foxp3^+^ (T reg cell) CD4^+^ T cell frequencies (B), and S1P1 expression levels (median) in T reg, Th1, and Th17 cells (C) were determined by flow cytometry. Data are representative of two independent experiments with five or six mice/group. **(D)** Tx-treated Atg5^WT^ and Atg5^ΔProx1^ mice were adoptively transferred with RORγt^GFP^FoxP3^RFP^ OT-II T cells and immunized with OVA_II_/CFA the day after. 5 d later, distances between RORγt-GFP^+^ Th17 cells and Lyve1^+^ LECs were calculated on LN sections, four or five mice/group. **(E)** SphK1, SphK2, and autophagosome (LC3-GFP^+^ organelles) staining in LECs sorted from LC3-GFP mice cultured and starved or not in HBSS+CQ for 4 h. Data are representative of three independent experiments with two or three mice/group. **(F)** SphK1 protein levels and LC3-II/LC3-I ratios (fold change) on sorted LECs from Tx-treated Atg5^WT^ and Atg5^ΔProx1^ mice, cultured and starved or not for 4 h. Data are representative of three independent experiments with two or three mice/group. **(G)** LECs sorted from Tx-treated Atg5^WT^ and Atg5^ΔProx1^ mice were cultured in the bottom part of a transwell membrane and starved or not for 16 h. Activated CD4^+^ T cells, treated or not treated with FTY-720, were added in the upper part. After 4 h, CD4^+^ T cell transmigration was analyzed by flow cytometry in the bottom part. Results are expressed as the ratio of migrated CD4^+^ T cells normalized to the control group (nonstarved WT LECs without FTY-720). Data are representative of three independent experiments with three or four mice/group. **(H)** S1P quantification (ELISA) from sorted Tx-treated Atg5^WT^ and Atg5^ΔProx1^ LECs cultured in RPMI with 1% of BSA (FBS free) for 48 h (two independent experiments with five or six mice/group). Unpaired *t* test (A, B, and H), one-way ANOVA (C, F, and G), and Kruskal Wallis (D). * P < 0.05, ** P < 0.01, and *** P < 0.001. Error bars correspond to lower and higher values for each group (A–D, F, and H) and to SEM (G).

**Figure S4.**
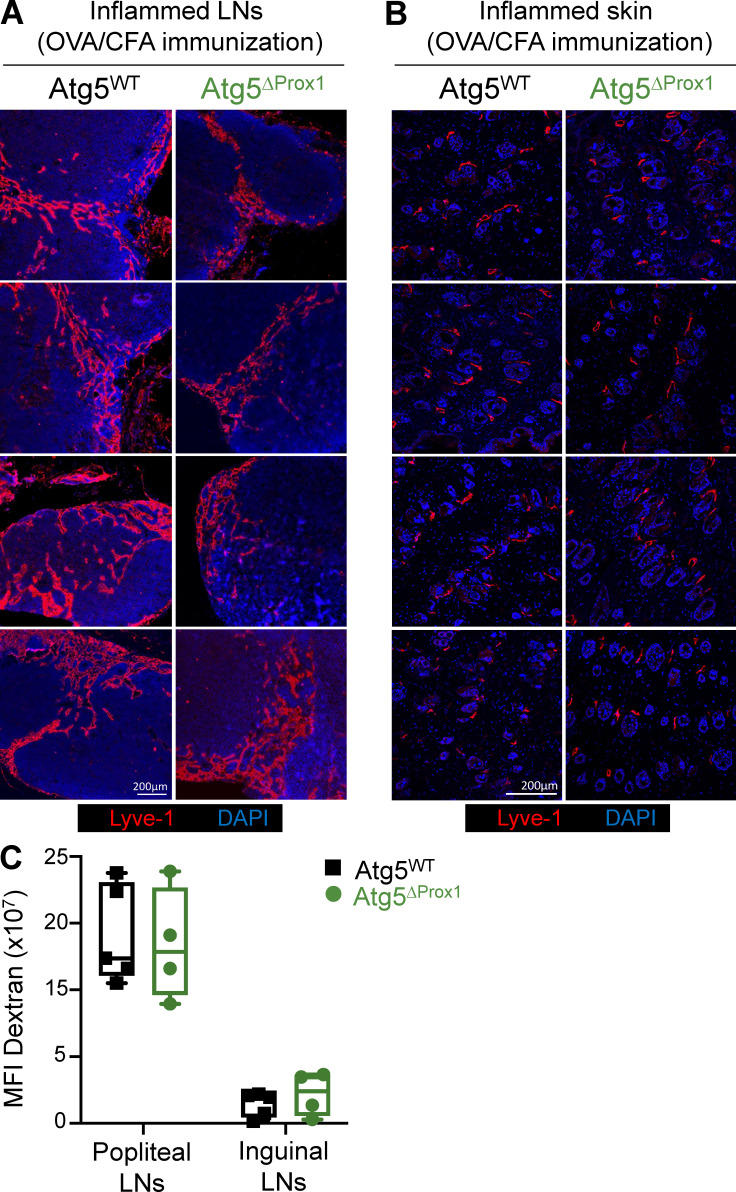
**Abolition of autophagy in LECs does not impair lymphatic vessel organization and draining functions upon inflammation. (A–C)** Tx-treated Atg5^WT^ and Atg5^ΔProx1^ mice were injected with OVA + CFA. 5 d later, lymphatic vessels (Lyve1^+^) were analyzed on sections from dLNs (A) and back skin (B). Images show organs from individual mice. **(C)** Dextran-FITC (40 kD) was injected in the left footpad of Atg5^WT^ and Atg5^ΔProx1^ mice 5 d after OVA + CFA injection. 30 min later, dextran-FITC mean fluorescence intensity (MFI) was measured in draining (popliteal and inguinal) LNs to evaluate lymphatic vessel draining functions. Right footpads were used as ipsilateral controls to remove background signal. Data are representative of two independent experiments, four or five mice/group each. **(C)** Unpaired *t* test. Error bars correspond to lower and higher values for each group.

One possibility is that our observations are consequence rather than the cause of more severe CIA in Atg5^ΔProx1^ mice. To determine whether inflammation-induced autophagy in LECs indeed regulates their ability to (1) promote T reg cells in LNs and (2) provide a local S1P gradient to modulate effector T cell egress from LNs, we performed experiments using a short-term induced inflammation model. Atg5^ΔProx1^ and Atg5^WT^ mice were s.c. immunized with OVA_II_ and CFA. 5 d later, recapitulating the results obtained with CIA mice, we observed a decrease of Th17 cell and T reg cell frequencies in dLNs of Atg5^ΔProx1^ mice, without any difference in Th1 cell frequency (Fig. 9, A and B). In addition, and similar to the CIA settings, Th17 cells expressed higher levels of S1P1 compared with both T reg and Th1 cells (Fig. 9 C). To assess the localization of Ag-specific Th17 cells in LNs, the same experiment was repeated by transferring OVA-specific TCR transgenic OT-II CD4^+^ T cells crossed with RORγt^+^ GFP mice 1 d before OVA_II_ + CFA immunization. 5 d after immunization, Th17 (RORγt-GFP^+^) OT-II cells localized significantly closer to medullar LECs (Lyve1^+^) in dLNs from Atg5^ΔProx1^ compared with Atg5^WT^ mice (Fig. 9 D). Altogether, these data demonstrate that upon short-term induced inflammation, LN LECs activate the autophagy pathway, leading to decreased T reg and Th17 cell frequencies, the latter likely being a consequence of impaired Th17 cell egress from inflamed LNs. We assessed whether the structure of LN or skin LECs or lymphatic vessel drainage functions could be altered under inflammatory settings by the loss of autophagy in LECs and play a role in our observations. Lyve-1 staining on sections of LN and skin from Atg5^ΔProx1^ and Atg5^WT^ immunized mice did not reveal any alterations of lymphatic vessel structure (Fig. S4, A and B). Furthermore, lymphatic drainage, measured by the flow out of Alexa Fluor 488–dextran from skin to dLNs, appeared similar in both Atg5^ΔProx1^ and Atg5^WT^ immunized mice (Fig. S4 C). Therefore, abrogation of autophagy in LECs does not affect their structure and drainage functions.

**Figure S5.**
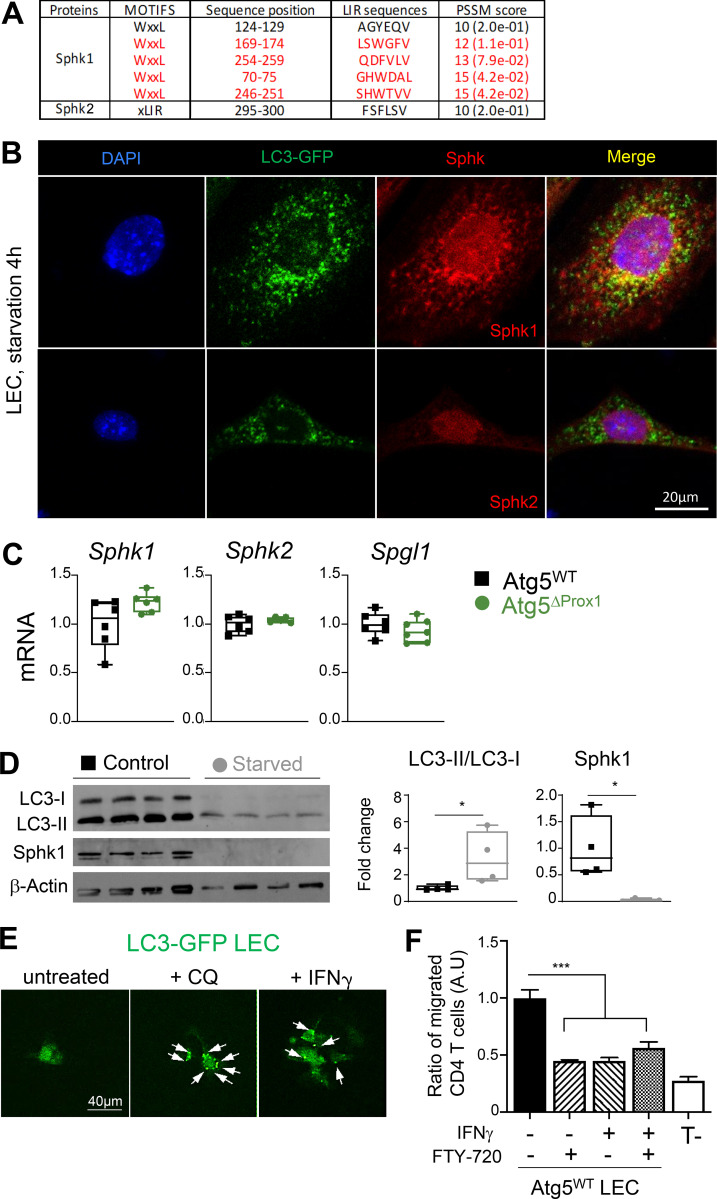
**Autophagy in LECs induces the degradation of SphK1. (A)** PSSM scores for SphK1 and SphK2 amino acid sequences. **(B)** SphK1, SphK2, and autophagosome (LC3-GFP^+^ organelles) staining in LECs sorted from LC3-GFP mice cultured and starved in HBSS + CQ for 4 h. Data are representative of three independent experiments with two or three mice/group. **(C)** mRNA levels of SphK1, SphK2, and SPGL1 (Sphingosine lyase 1) in LECs sorted from dLNs of Tx-treated Atg5^WT^ and Atg5^ΔProx1^ CIA mice at day 31 (six or seven mice/group). **(D)** Western blot showing SphK1 protein levels and LC3-II/LC3-I ratios in human iLECs, cultured and starved or not for 4 h. Data are representative of two independent experiments. LC3-I, 14 kD; LC3-II, 16 kD; β-actin, 43 kD; SphK1, ±70 kD. **(E)** LECs sorted from LN cultures of LC3-GFP mice were stimulated in vitro with either CQ or IFN-γ, and autophagosomes (LC3-GFP^+^ organelles) were assessed by microscopy. Arrows show the relocalization of LC3-GFP protein into autophagosome vesicles. Data are representative of two independent experiments. **(F)** LECs sorted from LEC/FRC cultures of Atg5^WT^ LEC mice were cultured in the bottom part of a transwell membrane and stimulated or not with IFN-γ (1 µg/ml) for 24 h. Activated CD4^+^ T cells treated or not with FTY-720 were added in the upper part. After 4 h, CD4^+^ T cell transmigration was analyzed by flow cytometry in the bottom part. Results are expressed as the ratio of migrated CD4^+^ T cells normalized to the control group (untreated WT LECs without FTY-720). Data are representative of four independent mice. Unpaired *t* test (C and D) and one-way ANOVA (F). * P < 0.05 and *** P < 0.001. Error bars correspond to lower and higher values for each group (C and D) and to SEM (F).

As LECs represent the main source of S1P production in LNs (Pham et al., 2010), we wondered whether autophagy in LECs directly impacts S1P production and consequently alters lymphocyte egress from LN. Two different enzymes, SphK1 and SphK2, respectively, are implicated in the generation of S1P. Analysis of amino acid sequences of SphK1 and SphK2 using the iLIR database software revealed that both enzymes exhibited LC3 interacting motifs. However, only SphK1 LIR motifs displayed a PSSM score >12, predicting an efficient targeting for SphK1, but not for SphK2, toward autophagosomes (Fig. S5 A). In vitro starvation of LC3-GFP LECs further showed that autophagosomes (LC3-GFP^+^) colocalized with SphK1 but not with SphK2 (Fig. 9 E and Fig. S5 B). Western blot analysis revealed that following starvation, Atg5^WT^ LECs exhibited higher levels of LC3-II, validating the induction of autophagosome formation (Fig. 9 F). Interestingly, this observation was associated with decreased SphK1 protein level in Atg5^WT^ LECs (Fig. 9 F), establishing a direct link between autophagy induction and SphK1 degradation in WT LECs, and supporting a negative regulation of S1P production by autophagy in LECs. In striking contrast, LC3-II induction or SphK1 reduction was absent in Atg5^ΔProx1^ LECs upon starvation, indicating that autophagy in LECs regulates SphK1 protein levels (Fig. 9 F). Transcriptional analysis of LECs sorted from LNs of CIA mice at day 31, which display high autophagy levels (Fig. 1), did not reveal any variation in the expression of genes implicated in S1P production (SphK1 and 2 kinases) or in S1P degradation (SPGL1 lyase; Fig. S5 C). Thus, reduced levels of SphK1 observed in Atg5^WT^ LECs upon starvation (Fig. 9 E) are a consequence of protein degradation and not a regulation of gene expression. Similar results were obtained after starvation of human intestinal LECs (iLECs). Although LC3 signal was lower in starvation condition (possibly a consequence of protein degradation following the activation of the autophagy pathway in these cells), the LC3-II/LC3-I ratio was increased and was associated with a total degradation of SphK1 (Fig. S5 D).

To determine whether this novel pathway in LECs has an impact on T cell migration toward S1P gradients, in vitro transmigration assays were performed using Atg5^ΔProx1^ or Atg5^WT^ LECs, starved or not, and activated CD4^+^ T cells, pretreated or not with FTY-720. Transmigration of activated CD4^+^ T cells was analyzed 4 h later by flow cytometry. FTY-720 treatment inhibited T cell transmigration toward Atg5^WT^ LECs, confirming that effector T cells are sensitive to S1P production by LECs (Fig. 9 G). Inhibition of T cell transmigration was also observed after starvation of Atg5^WT^ LECs, indicating that autophagy in LECs might impair their ability to induce T cell migration. Accordingly, starvation of Atg5^ΔProx1^ LECs did not compromise T cell transmigration (Fig. 9 G). We further validated these results using an inflammatory stimulus instead of starvation. LECs isolated from LC3-GFP mice treated with IFN-γ exhibit an activation of the autophagy pathway (Fig. S5 E). Therefore, we performed in vitro activated T cell transmigration assays by using IFN-γ–treated WT LECs. We confirmed that this inflammatory stimulus impairs S1P-mediated T cell transmigration toward LECs (Fig. S5 F). Altogether, these results show that autophagy regulates the ability of LECs to induce S1P-mediated T cell transmigration. In agreement with this hypothesis, amounts of S1P in supernatant from Atg5^ΔProx1^ LEC cultures were significantly increased compared with Atg5^WT^ LECs (Fig. 9 H). Altogether, our data demonstrate that autophagy alters the ability of inflamed LECs to promote effector T cell migration, and more particularly Th17 cells, in an S1P-dependent manner by regulating SphK1 but not SphK2 protein levels.

## Discussion

LECs are important contributors to the shaping of immune responses. In particular, by presenting endogenously expressed PTA to autoreactive T cells, they play a major role in maintaining peripheral T cell tolerance (Cohen et al., 2010; Lee et al., 2007; Magnusson et al., 2008; Nichols et al., 2007; Dubrot et al., 2014). Here, we show that LECs regulate the development of CIA in C57BL/6 mice and that autophagy plays a crucial role in LEC immunomodulatory functions. Disease-mediated inflammation activates the autophagy pathway in LECs, leading on the one hand to an adjustment of the T reg cell frequencies and CD103 expression and, on the other hand, to an inhibition of pathogenic Th17 egress from LNs and subsequent homing to inflamed joints.

Studies have described LECs as MHCII-restricted Ag-presenting cells impacting T reg cell proliferation and differentiation (Nadafi et al., 2020; Baptista et al., 2014; Dubrot et al., 2018). However, whether the autophagy pathway is active in LECs and implicated in LEC Ag-presenting functions is still unknown. Our results demonstrate that autophagy is actively induced in LECs in LNs draining the site of CIA induction. Autophagy abolition in LECs results in alterations in the T reg cell population in dLNs, with impaired proliferation and decreased CD103 expression. This effect was recapitulated in T reg cells from mice in which MHCII was deleted in LECs, suggesting an implication of the autophagy pathway in LECs in MHCII-restricted Ag-presenting LEC functions. Furthermore, the decrease in T reg cell proliferation, which occurs in both Atg5^ΔProx1^ and MHCII^ΔProx1^ mice, is another hallmark of T reg cell dysfunction. Indeed, the proliferative rate of T reg cells is increased during the autoimmune insult and support their suppressive functions (Attridge and Walker, 2014).

However, whereas LN T reg cells exhibit impaired suppressive functions in mice lacking MHCII expression by LECs, this was not the case in mice with conditional deletion of autophagy in LECs. Therefore, additional autophagy-independent pathways of MHCII-mediated Ag presentation are active in LECs during CIA and maintain T reg suppressive functions. In particular, in the absence of autophagy, compensatory intracellular degradation pathways are activated and can function as an alternative source of endogenous MHCII Ags, among them chaperone-mediated autophagy (Wang and Muller, 2015). In parallel, LECs can capture and process exogenous Ags (Kedl and Tamburini, 2015; Tamburini et al., 2014; Hirosue et al., 2014). In vivo*,* the expression of the integrin CD103 by LN T reg cells is decreased in mice in which MHCII or autophagy is abrogated in LECs. CD103 is important for T reg cell retention in inflamed sites through interaction with the E-cadherin expressed by other cells, including synoviocytes (Agace et al., 2000; Luan et al., 2018). Accordingly, decreased CD103 expression has been correlated to an impaired ability of T reg cells to localize at the site of infection and to dampen inflammation, while their intrinsic suppressive functions remained unchanged (Suffia et al., 2005). Therefore, the decrease of CD103 expression by T reg cells observed during CIA after the deletion of either autophagy or MHCII in LECs may impair their abilities to regulate self-reactive effector T cells in inflamed knees. Accordingly, we observed altered CD103 expression by T reg cells in arthritic joints of mice lacking autophagy in LECs.

Our data show that both autophagy and MHCII deletion in LECs result in T reg cell alterations. Future experiments will establish whether a direct link exists between these two pathways in LECs. Analyzing the MHCII peptide repertoire of LECs could in particular unravel the contribution of autophagy to their peptidome. However, limited numbers of LECs ex vivo will make such studies challenging.

Apart from their Ag-presenting functions, another way among others for LECs to control immune responses is to regulate lymphocyte trafficking. During CIA or after OVA_II_ immunization, Th17 cells but not Th1 cells were decreased in LNs from mice in which LECs cannot induce autophagy. This effect further correlates with enhanced Th17 accumulation in inflamed joints, demonstrating an aberrant migratory pattern of Th17 in CIA when LECs are deficient for autophagy. Interestingly, we identified LN Th17 cells as high expressors of S1P1, the receptor implicated in effector T cell egress (Garris et al., 2014), compared with Th1 and T reg cells, indicating differences in S1P sensitivity between distinct effector T cell subsets. Supporting our in vivo data, in vitro Th1 and Th17 cell migration assays toward S1P gradients revealed an increased Th17 migration (three- to fourfold change) compared with Th1 (twofold change; Seki et al., 2010). Consistently, studies have shown that S1P1 overexpression in T cells promotes Th17 differentiation (Lee et al., 2010; Huang et al., 2007).

The fact that autophagy in LECs regulates Th17 egress from LNs during CIA deepens our understanding of disease progression. CIA is a Th17-dependent disease, and treatment with anti–IL-17–depleting antibodies strongly dampens disease severity (Park et al., 2012). In agreement, we show that a dysregulation of Th17 effector cell trafficking significantly exacerbates the CIA clinical symptoms.

In addition to playing a role in Ag presentation, autophagy has also been shown to be implicated in lipid metabolism, in particular sphingolipids such as ceramide and sphingosine (Alexaki et al., 2014). Although S1P metabolism has been described as inducing autophagy (Mitroi et al., 2017), a potential regulation of S1P production by the autophagy pathway has not been reported so far. Previous studies revealed that the accumulation of sphingolipids in cells deficient for autophagy was due to the gathering of different enzymes implicated in their metabolism, such as the serine palmitoyltransferase (SPT) catalyzing the condensation of serine and palmitoyl-CoA, the first step to convert ceramide toward sphingosine (Alexaki et al., 2014; Havé et al., 2019). SPT can be degraded by autophagy in hepatocytes, whereas autophagy inhibition led to intracellular SPT accumulation (Alexaki et al., 2014). In agreement with these studies, we show that SphK1 and SphK2, two enzymes implicated in sphingolipid metabolism and precisely in S1P production, exhibit predictive LC3-interacting domains, suggesting that they might be actively degraded by the autophagy pathway. Furthermore, SphK1 displays a high LC3-interacting motif PSSM score, predicting its efficient targeting toward autophagosomes. Accordingly, we observed that SphK1, but not SphK2, is regulated by autophagy, which is not surprising also considering the localization of the two enzymes. SphK1 is located in the cytoplasm, allowing potential interactions with LC3 proteins. In contrast, SphK2 is preferentially contained in the nucleus and other intracellular organelles (Wattenberg, 2010; Siow et al., 2010). Although only SphK1 protein turnover can be regulated by autophagy, disruption of this pathway is sufficient to significantly inhibit SphK1 protein degradation and increase S1P production by LECs. In agreement, it has been shown that SphK1 deletion in mice significantly decreases the S1P gradient in blood, altering lymphocyte trafficking (Adamiak et al., 2017). In contrast, SphK2 deficiency results in an augmented circulating S1P gradient, likely due to either a compensatory effect by SphK1 or an increased S1P stabilization (Adamiak et al., 2017). Accordingly, autophagy deficiency stabilizes/accumulates SphK1 intracellular levels, leading to an increase of S1P production by LECs. In addition, SphK2 mRNA expression levels in LECs were not affected by the deletion of autophagy, indicating that the increase of S1P production by autophagy-deficient LECs is not a consequence of decreased SphK2 levels as described in SphK2-deficient mice (Adamiak et al., 2017). Interestingly, while the injection of SphK2-specific siRNAs in mice does not impact CIA, mice that received specific SphK1 siRNA developed attenuated CIA (Lai et al., 2009). Our data demonstrate that in inflammatory conditions such as CIA, autophagy induction in LN LECs promotes SphK1 degradation, leading to reduced S1P production in LNs and limited Th17 cell exit, and finally to dampened disease development.

Relevant for our study, mice deficient for Spns2 (Spinster homologue 2) exhibit reduced disease severity in several inflammation and autoimmune mouse models, such as experimental autoimmune encephalomyelitis, CIA, colitis, or airway inflammation (Donoviel et al., 2015). LECs are well described as the main source of S1P in LNs and regulate lymphocyte exit from LNs toward the periphery (Pham et al., 2010). Several LEC subsets were recently defined within the LNs, and one particular subset localized in the paracortical sinus close to the medulla expresses elevated levels of Spns2 (Fujimoto et al., 2020). Spns2 is a transporter expressed by LECs, required for S1P release in the lymph (Mendoza et al., 2012). Because of their localization and their enhanced ability to generate S1P gradient, this specific LEC subset may play a major role for lymphocyte egress from LNs. Future investigations will determine how autophagy is regulated in this particular subset and how it affects S1P production and effector T cell egress under pathological conditions. LECs in skin lymphatic vessels can also produce S1P (Petrova and Koh, 2018).

In conclusion, we show that in CIA, T reg (including CD103^+^ T reg cells) and Th17 cells are altered in LNs from mice lacking autophagy in LECs. However, the way autophagy in LEC regulates these two cellular components is likely to be different. S1P-blocking experiments demonstrate that the priming of T reg cells is impacted by the loss of autophagy in LECs. These results argue for a local role of autophagy in LECs in regulating T reg cells in LNs during CIA. In contrast, whereas Th17 cells in LNs from CIA Atg5^ΔProx1^ mice are decreased compared with control mice, the difference is abolished upon S1P blockade, indicating a role for autophagy in LECs in modulating Th17 cell exit from LNs and not their priming. Specifically, the effect on Th17 cell homing is related to the regulation of S1P production by autophagy in LECs. Regarding the latter mechanism, it is difficult to decipher the contribution of LECs in lymphatic vessels and LN LECs in our conditional knockout mice, in which LECs from both compartments were deleted for autophagy. Both lymphatic vessel and LN LECs can produce S1P and may possibly be modulated by autophagy. The possible regulation of the pathway by autophagy in LECs from one or the other compartment nevertheless brings new insight into the role of LECs in dampening T cell–mediated inflammatory diseases by modulating effector T cell egress from LNs and migration in inflamed tissues. The identification of autophagy as a crucial modulator of S1P production by LECs might be considered as a novel way to modulate unwanted T cell responses under inflammatory conditions.

## Materials and methods

### Mice

WT mice (Charles River), OT2 (Barnden et al., 1998), ProxCre^ERT2^ (from T. Makinen, Uppsala University, Uppsala, Sweden; Bazigou et al., 2011) crossed with MHCII^f/f^ (MHCII^ΔProx-1^; Dubrot et al., 2018), LC3-GFP (from N. Mizushima, University of Tokyo, Tokyo, Japan; Mizushima et al., 2004), Flt4cre^ERT2^ (from S. Ortega, Spanish National Cancer Research Center, Madrid, Spain; Stanczuk et al., 2015), and Foxp3^RFP^RORγt^GFP^ (from M. Lochner, Institute for Medical Microbiology and Hospital Epidemiology, Hannover Medical School, Hanover, Germany; Yang et al., 2016) mice were used between the ages of 8 and 12 wk. All mice had a pure C57BL/6 background and were bred and maintained under specific pathogen–free conditions at the animal facility of Geneva Medical School and at Charles River in France. All procedures were approved and performed in accordance with the guidelines of the animal research committee of Geneva. ProxCre^ERT2^Atg5^f/f^ (Atg5^ΔProx-1^), Flt4cre^ERT2^Atg5^f/f^ (Atg5^ΔFlt4^), Atg5^f/f^ control (Atg5^WT^), ProxCre^ERT2^MHCII^f/f^ (MHCII^ΔProx-1^), and MHCII^f/f^ control (MHCII^WT^) mice were treated i.p. with Tx (T5648; Sigma-Aldrich), 1 mg/mouse twice a day for 4 d. All procedures were performed 2 wk after the last Tx injection.

### CIA

CIA was induced in C57BL/6 mice by using an adapted protocol based on previous publication (Inglis et al., 2008). Briefly, an emulsion of chicken type II collagen (Col2, 4 mg/ml; Chondrex) and CFA (4 mg/ml; BD Bioscience) was injected i.d. at days 0 and 21 (boost injection) at the base of the tail (50 µl) and 1 cm upper in the back (50 µl). Paw thickness was measured using a caliper, and weights were followed once a week up to day 21 and then every 1–2 d from day 21. Caliper measures were used to determine clinical scores (normalized to day 0 for each mice). Scores from 0 to 3 (0: no inflammation; 1: slight swelling; 2: pronounced swelling; 3: ankylosis) were attributed for each paw, and clinical scores represent the sum of the scores for the four paws. For histological analysis, knees were fixed in 4% paraformaldehyde (PFA) for 24 h and decalcified in Tris-EDTA buffer for 6 wk. After paraffin embedment, 5-µm sections were stained either with hematoxylin and eosin or with toluidine blue and blind graded. Histological analysis (scores from 0 to 3) was determined for each knee based on inflammation levels. Global inflammation scores represent the sum of the scores of two knees for each mouse. At least three sections per knee were examined to ensure extensive evaluation of the arthritic joints. The lesions were blindly evaluated for each joint as previously described using a four-point scale (0–3, where 0 is normal and 3 is severe; Biton et al., 2016; Clavel et al., 2006). This global histological score reflects both synovitis (synovial proliferation, inflammatory cell infiltration) and joint destruction (bone and cartilage thickness and irregularity and presence of erosions). We also separately evaluated articular destruction by considering the degradation of bone and cartilage regardless of inflammation on a four-point scale (0–3, where 0 is no destruction and 3 is the presence of subchondral bone erosions).

In some CIA experiments, FTY-720 (20 µg/mice; Sigma-Aldrich) was injected i.p. every 2 d from day 20 to day 30. For some other CIA experiments, anti-CD25–depleting antibody (100 µg/mice; Bioxcell) was injected i.p. every 3 d from day 19 to day 40.

### OVA_II_ immunization

Mice were immunized with an emulsion of CFA and OVA_II_ peptide (ISQAVHAAHAEINEAGR, 25 µg/50 µl; PolyPeptide) s.c. in each flank. 5 d later, cells from dLNs were isolated for FACS analysis.

For LN histology, Atg5^ΔProx1^ and Atg5^WT^ mice were injected i.v. with 10^6^ naive CD4^+^ T cells isolated from OT-II/Foxp3^RFP^RORγt^GFP^ mice and immunized 24 h later with OVA_II_ peptide emulsified in CFA (as described previously). 5 d after immunization, dLNs were frozen, and 5-µm-thick sections were fixed in PFA 4% at room temperature (RT) during 20 min. After washing, samples were permeabilized with Triton X-100 (1/400e) and blocked with normal goat serum (1/100e) for 30 min at RT and Triton X-100 (1/800e) for another 30 min at RT. Staining was performed overnight at 4°C using primary anti-GFP (1/1,000e; Thermo Fisher Scientific) and anti-Lyve1 (1/200e) antibodies. After washing, secondary antibodies against chicken Alexa Fluor 488 for GFP staining and anti-rabbit Alexa Fluor 546 (1/1,000e; Thermo Fisher Scientific) were used for 2 h at RT (in the dark). Samples were washed and stained for 5 min with DAPI. Pictures were acquired using a confocal microscope (LSM700; Zeiss). Distances between LECs (Lyve-1^+^ cells) and CD4^+^ OT-II Th17 cells (RORγt-GFP^+^ cells) were blinded and calculated using ImageJ software.

### Cell isolation

dLNs were cut into small pieces and digested in RPMI containing 1 mg/ml collagenase IV (Worthington Biochemical Corporation), 40 µg/ml DNase I (Roche), and 2% FBS for 30 min at 37°C. Remaining tissue pieces were further digested with 1 mg/ml collagenase D and 40 µg/ml DNase I (Roche) and 1% of FBS for 20 min at 37°C. The reaction was stopped by adding 5 mM EDTA and 10% BSA. Samples were further disaggregated through a 70-µm cell strainer, and Fc receptors were blocked with anti-CD16/32 antibody. For LNSC analysis, single-cell suspensions were magnetically enriched in CD45^neg^ cells by using CD45 microbeads (Miltenyi Biotec) according to the manufacturer’s instructions.

To assess joint infiltrating T cells, posterior legs from CIA mice were dissected. Muscles and skin were totally removed from the legs and paws, and femurs were dissociated from tibias. Full bones (femurs and tibia with paws) were digested in 5 ml of RPMI containing 1 mg/ml of collagenase A and 40 µg/ml of DNase I for 45 min at 37°C under gentle agitation. The reaction was stopped by adding 5 mM EDTA and 10% BSA, and cell suspensions were filtered (70 µm). A percoll 70–40% at 700 *g* for 25 min at RT was performed, and the cells at the interface between percoll 70% and 40% were harvested for flow cytometry analysis.

### In vitro suppression assay

2 wk after Tx treatment, mice were irradiated twice a day (500 mGy). The day after irradiation, 5 × 10^6^ BM cells from Foxp3^RFP^RORγt^GFP^ mice were injected (i.v.), and the mice received antibiotics in water for the next 2 wk. CIA was induced in BM chimeric mice after 8 wk, and T reg cells from dLNs were sorted (based on RFP expression) after 26 d with Aria sorter (BD Bioscience). T reg cells were co-cultured at different ratios with purified CTV-labeled naive CD25^neg^CD4^+^ T cells (magnetic beads isolation kit; Miltenyi Biotec) stimulated with anti-CD3 antibody (0.25 µg/ml; eBioscience), and BM-derived DCs were differentiated during 9 d with GM-CSF as described previously (Lippens et al., 2018). 3 d after, naive T cell proliferation was assessed by flow cytometry, and the proliferation index was calculated using FlowJo software. The division index (FlowJo software) provides the average number of cell divisions that a cell in the original population has undergone. This is an average even for cells that never divided (i.e., it includes the undivided peak).

### Stromal cell cultures

LEC and FRC cultures were performed as previously described (Fletcher et al., 2010). Briefly, LNs from one to three mice were dissected and digested with a freshly made enzymatic solution composed of RPMI-1640 containing 0.8 mg/ml Dispase, 0.2 mg/ml Collagenase P, and 0.1 mg/ml DNase I (Roche). Tubes were incubated at 37°C in a water bath and gently inverted at 10-min intervals to ensure the contents were mixed. Every 10 min, LNs were very gently mixed using a 1-ml pipette. Large fragments were allowed to settle before replacing the supernatant for fresh digestion mix. Supernatant containing the cells was kept in cold FACS buffer (2% FCS, 0.5% BSA, and 5 mM EDTA in PBS). These steps were repeated every 10 min until complete digestion. Cells were washed, filtered (70 µm), and incubated in 6-well plates coated with human fibronectin and collagen for 30 min at 37°C at the concentration of 7 × 10^6^ cells/well in MEM supplemented with 10% batch-tested, low-Ig FCS and 1% penicillin/streptomycin. Plates were washed, and culture medium was renewed every day to remove nonadherent CD45^+^ cells. After 5 d, cultures primarily contained a mixture of LECs and FRCs. Autophagosome formation was assessed in LECs and FRCs sorted from LC3-GFP mice, cultured, and stimulated with either IFN-γ (1 µg/ml) or CQ (50 µM) for 24 h. Then, organelle formation (LC3-GFP^+^) was determined following LC3 relocalization into the cells.

### Western blot

After stromal cell culture using LNs from Atg5^ΔProx1^ and Atg5^WT^ mice, LECs were sorted by flow cytometry (Astrios sorter; Beckman Coulter) and cultured (100,000 cells/well) in 12-well plates coated as before. At cell confluence, LECs were starved (HBSS) for 4 h to induce autophagy. Proteins from LECs (starved or not) were extracted using a lysis buffer (Tris, pH 8, 150 mM, NaCl 50 mM, and 1% NP-40) with protease inhibitor (Millipore). Proteins were quantified, and 5 µg was warmed at 95°C for 5 min and migrated onto a 12% SDS-acrylamide gel. After transfer on a polyvinylidene difluoride membrane, a saturation step was performed with PBS containing 5% milk for 1 h at RT, and anti-LC3 (1/2,000e; Cell Signaling) or anti-SphK1 (1/1,000e; BIOSS USA) antibodies were incubated overnight at 4°C under agitation. After washing, the membrane was incubated with secondary antibodies, goat anti-rabbit–HRP (1/10,000e; Bio-Rad), or anti-mouse–HRP (1/5,000e; Santa Cruz) for 1 h at RT, and the HRP substrate (Millipore) was used to reveal the proteins. Protein expression levels were normalized to GAPDH control protein expression level.

Human iLECs (provided by T. Petrova, University of Lausanne, Lausanne, Switzerland; Norrmén et al., 2010) were seeded in a 12-well plate coated with human fibronectin in EGM-2 medium (Lonza; Ruwag) at a concentration of 100,000 cells in 2 ml medium. At confluence, cells were starved in HBSS for 4 h, and Western blots were performed as described above.

### In vitro transmigration assay

LECs were sorted by flow cytometry (Astrios sorter; Beckman Coulter) from Atg5^ΔProx1^ and Atg5^WT^ stromal cell cultures and incubated (100,000 LECs/well) in 24-well plates coated as before. At cell confluence, cells were starved (in HBSS) or not starved overnight (250 µl). LEC culture medium was supplemented with 600 µl of RPMI, and transwell membrane (5-µm pores; Cornix) was added in each well. CD4^+^ T cells were purified from naive mice using a magnetic isolation kit (Miltenyi Biotec) and activated in vitro (anti-CD3 + anti-CD28 antibodies) for 3 d. Then, activated CD4^+^ cells were treated or not with FTY-720 (100 nM; Sigma-Aldrich) for the last 12 h, washed, and added (150,000 cells) in the upper part of the transwell. 4 h later, CD4^+^ T cell migration was analyzed in the bottom part by flow cytometry using counting beads (BD Bioscience). Results are expressed as ratio of CD4^+^ T cell migration compared with control group (unstarved Atg5^WT^ LECs with CD4^+^ T cells untreated with FTY-720).

### S1P ELISA

LECs were sorted by flow cytometry (Astrios sorter; Beckman Coulter) from LEC/FRC cultures of Atg5^ΔProx1^ and Atg5^WT^ LNs, and 100,000 cells/well were seeded in 12-well plates coated as before. At confluence, LECs were washed in PBS and cultured in RPMI (FBS free) containing 1% penicillin/streptomycin and 1% BSA. After 48 h, supernatants were harvested and centrifuged at 10,000 *g* for 10 min to eliminate cell debris. Quantification of S1P in supernatants was performed using the General Sphingosine-1-Phosphate (S1P) ELISA kit (MyBioSource) according to the manufacturer’s instructions.

### LIR database analysis

For each protein, amino acid sequences were analyzed using a published iLIR database (Nezis, 2021) to obtain predictive LIRs and their PSSM scores. Predictive LIRs with PSSM scores >12 give reliable prediction for targeting protein toward autophagosomes (with an accuracy >75.4% and sensitivity >88.9%; Kalvari et al., 2014).

### Flow cytometry

Anti-CD45 (30-F11), anti-gp38 (8.1.1) and anti-CD31 (390), anti-CD8 (53–6.7), anti-CD25 (PC61), FoxP3 (FJK-16S), anti-Ki67 (SolA15), anti-IL-17 (eBio17B7), and anti–IFN-γ (XMG1.2) were from eBioscience and/or Invitrogen. Anti-TCRb (H57-597), anti-CD4 (GK1.5), anti–I-Ad/I-Ed (2G9), and anti-CD103 (M290) were from BD Bioscience, and anti-S1P1 (FAB7089P) was from R&D Systems. LC3 staining was performed using the “Flowcellect Autophagy LC3 Antibody-based assay kit” (FCCH100171; Millipore) according to the manufacturer’s instructions.

For LNSC flow cytometry sorting, ex vivo–enriched CD45^neg^ LN cells or LEC/FRC cultures were stained with antibodies against CD45, gp38, and CD31, and cells were sorted using an Astrios (Beckman Coulter).

For intracellular cytokine staining, cells were stimulated for 4 h in vitro with PMA/ionomycin (Sigma-Aldrich) and GolgiPlug (BD Bioscience) in RPMI containing 10% heat-inactivated FBS, 50 mM 2-mercaptoethanol, 100 mM sodium pyruvate, and 100 µM penicillin/streptomycin at 37°C and 5% CO_2_. FoxP3, IL-17, and IFN-γ were stained using fixation/permeabilization buffer (eBioscience) according to the manufacturer’s instructions. Cells were acquired on Fortessa (BD Bioscience), and analysis was performed using FlowJo software.

### Immunofluorescence

#### Staining for autophagosomes and MHCII

LC3-GFP mice were injected s.c. with IFN-γ (1 µg/ml) and with CQ 24 h later (50 µM) in both flanks and neck area or immunized for CIA. Stromal cells (LECs, BECs, and FRCs) from dLNs were sorted (Astrios; Beckman Coulter) after 24 h. Sorted cells were cytospined and fixed in acetone at −20°C for 20 min. After washing, samples were permeabilized with Triton X-100 (1/400e) and blocked with normal goat serum (1/100e) for 30 min at RT and Triton X-100 (1/800e) for another 30 min at RT. Staining was performed overnight at 4°C using primary anti-GFP (1/1,000e; Thermo Fisher Scientific) and anti–MHC-II biotinylated (1/200e) in some experiments. After washing, secondary antibodies against chicken Alexa Fluor 488 for GFP staining and streptavidin Alexa Fluor 546 for MHCII detection (1/300e; eBioscience) were incubated for 2 h at RT (in the dark).

#### Staining for SphK and autophagosomes

LECs sorted from LNs of LC3-GFP mice were starved (HBSS, devoid of FBS) and treated with CQ (50 µM) for 4 h to induce and stabilize autophagosomes. Then, LECs were fixed in PFA 4% at RT for 20 min. After washing, samples were permeabilized with Triton X-100 (1/400e) and blocked with normal goat serum (1/100e) for 30 min at RT and with Triton X-100 (1/800e) for another 30 min at RT. Staining was performed overnight at 4°C using primary anti-GFP (1/1,000e; Thermo Fisher Scientific) and anti-SphK1 or anti-SphK2 (1/200e; Bioss USA) antibodies. After washing, secondary antibodies against chicken Alexa Fluor 488 for GFP staining and anti-rabbit Alexa Fluor 546 (1/1,000e; Thermo Fisher Scientific) were incubated for 2 h at RT (in the dark).

#### Staining of lymphatic vessels in skin and LNs

Tissues were harvested and frozen 5 d after OVA + CFA immunization, and 5-µm-thick sections were fixed in PFA 4% at RT during 20 min. Staining was performed as described previously with anti-Lyve1 antibody (1/400e). After washing, secondary anti-rabbit Alexa Fluor 546 antibody (1/1,000e; Thermo Fisher Scientific) was incubated for 2 h at RT (in the dark).

In all cases, samples were washed and stained for 5 min with DAPI. Pictures were acquired using a confocal microscope (LSM700; Zeiss).

#### Staining of T and B cell zones in LNs

Skin LNs were harvested and frozen 2 mo after Tx treatment. 5-µm-thick sections were fixed in PFA 4% at RT during 20 min. After washing, samples were permeabilized with Triton X-100 (1/400e) and blocked with normal goat serum (1/100e) for 30 min at RT and with Triton X-100 (1/800e) for another 30 min at RT. Staining was performed overnight at 4°C using anti–B220–Alexa Fluor 488 (eBioscience clone RA3-6B2, coupled to Alexa Fluor 488 1/50e) and anti-CD3 Alexa Fluor 647 (clone 145-2C11, 1/100e; Biolegend) antibodies. After washing and staining for 5 min with DAPI, pictures were acquired using an upright spinning disk confocal microscope (Axio Examiner Z1 Advanced Microscope Base; Zeiss) equipped with a confocal scanner unit CSU-X1 A1 (Yokogawa Electric Corporation). The fluorescence was detected with an electron-multiplying charge-coupled device camera (Evolve 512 10 MHz Back Illuminated; Photometrics) and a 10×/0.3 numerical aperture water immersion objective (W Plan Apochromat; Zeiss) using three lasers with the excitation wavelengths of 405 nm, 488 nm, and 640 nm (LaserStack v4 Base, 3i) and appropriate band-pass-emission filters (Semrock). Three-dimensional image stacks were obtained by sequential acquisition of multiple fields of views along the z axis using a motorized XY-stage (ProScan; Prior). SlideBook software (6.0.17, 3i) was used for image acquisition, and the subsequent deconvolution, creation of maximum projection, and generation of montage images from contiguous positions were performed using the Fiji plugins Deconvolutionlab2 and grid/collection stitching (Sage et al., 2017; Preibisch et al., 2009).

### Lymphatic vessel drainage

5 d after OVA + CFA immunization (s.c., tail base), dextran–Alexa Fluor 488 (40 kD) was injected in the left footpad (50 µl at 5 mg/ml). 30 min later, dLNs (popliteal and inguinal) from the left side and their ipsilateral control from the right side were harvested and dissociated in 200 µl of PBS. A fluorospectrometer (Spectamax Paradigm; Molecular Devices) was used to determine Alexa Fluor 488 fluorescence intensity in dLNs. Background of fluorescence in the ipsilateral side was removed to the intensity of the dextran–Alexa Fluor 488 left side.

### Quantitative RT-PCR

Total RNA was isolated using RNeasy kit (Qiagen) for LECs, BECs, and FRCs from different samples. For mRNA extraction from paws and dLNs, a homogenizer (Polytron) was used to dissociate the tissues in Tri-Reagent (Ambien). cDNA was synthesized using random hexamers and M-MLV reverse transcription (Promega). PCRs for cDNAs were performed with the CFX Connect real-time PCR detection system and iQ SYBR green super mix (Bio-Rad Laboratories). Results were normalized to GAPDH cDNA expression level.

Primer sequences: Gpa-A33, F: 5′-CCG​AAG​TCA​GAC​GGA​AAG​AG-3′ and R: 5′-TGC​TGG​AGG​TGC​AGA​TGT​AG-3′; PLP, F: 5′-CAG​GGG​GCC​AGA​AGG​GGA​GG-3′ and R: 5′-GCA​GCC​CCA​CAA​ACG​CAG​C-3′; Tyr, F: 5′-GCC​CAG​CAT​CCT​TCT​TC-3′ and R: 5′-TAG​TGG​TCC​CTC​AGG​TGT​TC-3′; NTS, F: 5′-CAG​CTC​CTG​GAG​TCT​GTG​CT-3′ and R: 5′-GAG​TAT​GTA​GGG​CCT​TCT​GGG-3′; Amy2, F: 5′-TGG​CGT​CAA​ATC​AGG​AAC​ATG-3′ and R: 5′-AAA​GTG​GCT​GAC​AAA​GCC​CAG-3′; SEMA3D, F: 5′-GAC​CAT​GTT​GTT​TCT​TCC​AGT​CA-3′ and R: 5′-CAG​TCC​TTC​TGA​TGA​ACC​CAA​A-3′; col2a1, F: 5′-CAT​TGT​TGG​TCT​GCC​TGG​TC-3′ and R: 5′-TCT​CAC​CAC​GAT​CTC​CCT​TG-3′; SphK1, F: 5′-CTC​CTG​GGC​AAC​ACC​GAT​AA-3′ and R: 5′-ATG​GTT​CTT​CCG​TTC​GGT​GA-3′; SphK2, F: 5′-ACA​GAC​AGA​ACG​ACA​GAA​CCA-3′ and R: 5′-CAG​GTC​AAC​ACC​GAC​AAC​CT-3′; SPGL1, F: 5′-AGC​TCC​ATG​GAT​GGT​TCC​TG-3′ and R: 5′-TAA​CGC​CAA​GTC​CCG​GTA​AG-3′; mSAA, F: 5′-GTT​CAC​GAG​GCT​TTC​CAA​GGG​GCT​GG-3′ and R: 5′-CCT​GAA​AGG​CCT​CTG​TTC​CAT​CAC​TG-3′; IL-6, F: 5′-TCT​GTA​TCT​CTC​TGA​AGG​ACT​CTG​GCT-3′ and R: 5′-TCA​ACA​ACG​ATG​ATG​CAC​TTG​CAG​A-3′; and GAPDH, F: 5′-CCC​GTA​GAC​AAA​ATG​GTG​AAG-3′ and R: 5′-AGG​TCA​ATG​AAG​GGG​TCG​TTG-3′.

### Statistical analysis

Statistical analysis was performed using GraphPad prism software. One-way ANOVA, two-way ANOVA, unpaired *t* test (Mann-Whitney), Kruskal-Wallis test, or multiple *t* test comparisons corrected with Sidak-Bonferroni method was performed to determine the P value: * for P < 0.05, ** for P < 0.01, and *** for P < 0.001. For graphs representing violin plots, error bars correspond to lower and higher values for each group. For the rest of the analysis (normal histograms, CIA clinical scores, and T reg cell suppression), error bars correspond to SEM.

### Online supplemental material

Fig. S1 shows LN B cell, natural killer, neutrophil, macrophage, and DC frequencies and their expression levels of the costimulatory molecules CD80 and CD86; B and T cell areas of LN sections; and LN weights from Atg5^ΔProx1^ and Atg5^ΔWT^ mice at steady state. Fig. S2 shows T cell frequencies in secondary lymphoid organs as well as peripheral tissues at steady state. Fig. S3 provides T reg cell analysis in dLNs from CIA MHCII^ΔProx1^ and MHCII^WT^ mice at days 26 and 31. Fig. S4 shows lymphatic vessel organization in dLNs and skin, as well as lymphatic drainage functions, of Atg5^ΔProx1^ and Atg5^WT^ mice immunized with OVA/CFA. Fig. S5 shows the iLIR motifs for SphK1 and SphK2; SphK1, SphK2, and SPGL1 mRNA expression levels in LN LECs of arthritic Atg5^ΔProx1^ and Atg5^WT^ mice; the Western blot of LC3-II/I; SphK1 in iLECs starved or not starved; and the autophagosome formation (LC3-GFP^+^ organelles), induced after IFN-γ treatment, in cultured LECs and their ability to induce T cell transmigration under inflammatory stimulus (IFN-γ).

### Data availability

The data underlying this article are accessible at https://doi.org/10.26037/yareta:oijq3aigebfctoioe3mgs2s4am.
